# Hi-C chromosome conformation capture sequencing of avian genomes using the BGISEQ-500 platform

**DOI:** 10.1093/gigascience/giaa087

**Published:** 2020-08-26

**Authors:** Marcela Sandoval-Velasco, Juan Antonio Rodríguez, Cynthia Perez Estrada, Guojie Zhang, Erez Lieberman Aiden, Marc A Marti-Renom, M Thomas P Gilbert, Oliver Smith

**Affiliations:** Section for Evolutionary Genomics, The GLOBE Institute, University of Copenhagen, Øster Farimagsgade 5A, 1353 Copenhagen, Denmark; CNAG-CRG, Centre for Genomic Regulation, Barcelona Institute of Science and Technology, Baldiri i Reixach, 4-8, 08028 Barcelona, Spain; Center for Genome Architecture, Department of Molecular and Human Genetics, Baylor College of Medicine, Houston, TX 77030, USA; China National GeneBank, BGI-Shenzhen, Shenzhen 518083, China; Center for Genome Architecture, Department of Molecular and Human Genetics, Baylor College of Medicine, Houston, TX 77030, USA; Center for Theoretical Biological Physics, Rice University, Houston, TX 77005, USA; Broad Institute of the Massachusetts Institute of Technology and Harvard University, Cambridge, MA, USA; Department of Computer Science and Computational Applied Mathematics, Rice University, 6100 Main St, Houston, TX 77005-1827, USA; CNAG-CRG, Centre for Genomic Regulation, Barcelona Institute of Science and Technology, Baldiri i Reixach, 4-8, 08028 Barcelona, Spain; Centre for Genomic Regulation, Barcelona Institute for Science and Technology, Doctor Aiguader 88, Barcelona 08003, Spain; Pompeu Fabra University, Doctor Aiguader 88, Barcelona 08003, Spain; ICREA, Pg. Lluís Companys 23, 08010 Barcelona, Spain; Section for Evolutionary Genomics, The GLOBE Institute, University of Copenhagen, Øster Farimagsgade 5A, 1353 Copenhagen, Denmark; Norwegian University of Science and Technology, University Museum, 7491 Trondheim, Norway; Section for Evolutionary Genomics, The GLOBE Institute, University of Copenhagen, Øster Farimagsgade 5A, 1353 Copenhagen, Denmark; Micropathology Ltd, University of Warwick Science Park, Coventry CV4 7EZ, UK

**Keywords:** Hi-C, BGISEQ-500, next-generation sequencing, chromosome conformation capture

## Abstract

**Background:**

Hi-C experiments couple DNA-DNA proximity with next-generation sequencing to yield an unbiased description of genome-wide interactions. Previous methods describing Hi-C experiments have focused on the industry-standard Illumina sequencing. With new next-generation sequencing platforms such as BGISEQ-500 becoming more widely available, protocol adaptations to fit platform-specific requirements are useful to give increased choice to researchers who routinely generate sequencing data.

**Results:**

We describe an *in situ* Hi-C protocol adapted to be compatible with the BGISEQ-500 high-throughput sequencing platform. Using zebra finch (*Taeniopygia guttata*) as a biological sample, we demonstrate how Hi-C libraries can be constructed to generate informative data using the BGISEQ-500 platform, following circularization and DNA nanoball generation. Our protocol is a modification of an Illumina-compatible method, based around blunt-end ligations in library construction, using un-barcoded, distally overhanging double-stranded adapters, followed by amplification using indexed primers. The resulting libraries are ready for circularization and subsequent sequencing on the BGISEQ series of platforms and yield data similar to what can be expected using Illumina-compatible approaches.

**Conclusions:**

Our straightforward modification to an Illumina-compatible *in situ*Hi-C protocol enables data generation on the BGISEQ series of platforms, thus expanding the options available for researchers who wish to utilize the powerful Hi-C techniques in their research.

## Background

Determining the organization of chromatin and chromosomes within a cell nucleus carries great potential for describing genome function, regulation, interactions, and evolution. Advancements in molecular techniques since the beginning of the 21st century have allowed steady progress in uncovering these interactions using various forms of DNA-DNA proximity ligation [1] and chromosome conformation capture (3C) techniques [2]. These methods involve the covalent cross-linking of chromatin regions interacting in space, followed by restriction enzyme digestion and proximity ligation of fragments from interacting regions. This is then followed by DNA sequencing, yielding data that can be used to describe proximal interactions between distant genomic loci [3]. Earlier versions known as 3C and 4C generally focused on single or a few loci, using locus-specific primers and Sanger sequencing [4, 5]. Later, multiple parallel loci contacts could be quantified at once using either quantitative PCR approaches, microarray characterization, or early next-generation sequencing (NGS) platforms such as Roche 454, in a protocol known as 5C [6, 7].

More recently, however, NGS methods coupled with tailored bioinformatic tools have allowed a full range of interactions to be described at the genome scale using a method known as Hi-C [4]. These advances allow for insights not only into overall chromatin structure [8] but into the interactions between genes and their regulatory elements [9, 10] and their functions [11], revealing further layers of epigenomic activity to be decoded [12]. These genome-scale Hi-C analyses have used established NGS platforms as the preferred sequencing method, particularly those with short-read characteristics. Indeed, paired-end (PE) sequencing offered by the Illumina platforms [13] is widely used in Hi-C owing to their ubiquity and relatively universal final library constructs. In Hi-C, the mapping of both ends of a single read reveals a high sequential distance but at the same time implies a close physical proximity of the 2 ends, allowing for the identification of chromosomal contacts. While long-read platforms such as Pacific Biosciences are invaluable for *de novo* sequencing approaches owing to their ability to overcome nebulous genomic regions such as short tandem repeats or AT-rich regions, they cannot provide details of the structure of a genome. Therefore, for the time being, short, PE reads are the sequencing method of choice for identifying spatially close loci using 3C-based methods.

Although Illumina platforms have dominated the sequencing for these type of methods, alternative technologies are now appearing. One of these is the BGISEQ series of platforms, whose general workflow and stepwise sequencing procedures are similar to those of Illumina series, yet the sequencing templates have marked differences. Illumina uses hybridization clustering followed by bridge amplification [13], whereas the BGISEQ technology combines DNA nanoball nanoarrays [14] with polymerase-based stepwise sequencing. During this process, also called nanoball sequencing, the DNA undergoes an iterative ligation to circularize the DNA molecules, which are then replicated for the generation of DNA nanoballs. This iterative process generates billions of DNA nanoballs from each DNA molecule that are then loaded into a flow cell and sequenced [15]. The BGISEQ has several features that have proven attractive to researchers within different fields. Namely, it allows several sequencing read lengths (50, 100, 150 bp) either for single read or PE sequencing; it has a very high throughput where ≥2 billion PE reads per flow cell are generated in only a few days; and it allows an easy adaptation of library construction protocols. Comparisons between the Illumina HiSeq-2500 and BGISEQ-500 platforms have been made previously for the sequencing of shotgun DNA [16] and RNA [17], with both studies finding very little difference between platforms for standard metrics for each molecule type such as clonality, endogenous content, GC content, and sequence quality scores. Furthermore, the Illumina HiSeq-4000 and BGISEQ-500 have also been compared for sequencing of exomes [18] and transcriptomes [19], validating the capability of BGISEQ-500 to be established as a competitive and reliable platform for both exome and transcriptome analysis.

Given that the performance of the BGISEQ-500 as a sequencer is similar to Illumina, we explored whether the Hi-C method could be adapted to the BGISEQ-500 platform. Specifically we adapted the *in situ* Hi-C protocol published by Rao et al. [8] by changing the adapter ligation step to use blunt-end ligation, meaning omission of the A-tailing step in Illumina library construction. Additionally, we introduced a post-ligation fill-in step to remove overhangs present on the distal ends of the BGISEQ adapters. We also modified the sequences of the adapters and amplification primers to be compatible with the BGISEQ-500 platform. We then submitted amplified libraries to BGI-Europe for circularization, DNA nanoball construction, and sequencing, and analysed the data to assess the method's performance.

## Results

We processed 3 different zebra finch (*Taeniopygia guttata*) tissue samples using the BGI-Hi-C protocol developed for this study and described in detail in the Supplementary Materials and Methods. The BGI-Hi-C libraries were quantified and visualized using a BioAnalyzer Instrument (Supplementary Fig. S1), pooled and sequenced on a partial lane of the BGISEQ-500 with PE100 sequencing mode. Sequencing reads were analysed using TADbit [20], a pipeline developed for Hi-C experiments to pre-process the reads, assess the quality of the Hi-C experiments (Supplementary Figs S2 and S3), map the reads to a reference genome, filter and normalize interaction data, analyse the resulting interaction matrices, and generate statistics and maps to model and explore 3C-based data.

The 3 zebra finch samples yielded ~29 million, ~3 million, and ~2 million PE sequence reads, respectively (Table 1). Using the TADbit pipeline we mapped and filtered all reads for the 3 samples (Table 2). Mapping was performed following a fragment-based method implemented in TADbit [20]. To map the sequence data we used the available reference genome on NCBI for the zebra finch (GCF_000151805.1_Taeniopygia_guttata-3.2.4).

**Table 1:** TADbit mapping and quality statistics of the Hi-C-BGI experimental results

				Out of 100,000 reads			Total No. interactions; both reads mapped (% initial)
Sample	Index	Read	Initial reads	Digested sites (%)	Reads with ligation site (%)	Uniquely mapped pairs (% initial)	
Oz13	10	1	29,436,352	83.6	31.6	18,670,087 (63.2%)	18,269,316 (62.1%)
		2	29,436,352	80.4	29.8	18,127,333 (61.6%)	
Mz13	18	1	3,069,136	68.5	14.8	2,396,713 (78.1%)	2,201,431 (71.7%)
		2	3,069,136	66.2	14.4	2,297,654 (74.9%)	
Mz17	19	1	2,274,286	44.8	7.8	1,711,896 (75.3%)	1,554,764 (68.4%)
		2	2,274,286	42.5	7.3	1,673,397 (73.6%)	

**Table 2:** For each of the 3 experiments, reads lost after each of the filters applied, approximate comparison to numbers expected for a standard Illumina experiment, and final number of valid pairs considered

Filter type	Illumina expected % (for MboI)	Oz13	%	Mz13	%	Mz17	%
Self-circle	<1	11,385	0.06	540	0.02	440	0.03
Dangling end	2.3–58.4	6,072,260	33.2	934,066	42.4	781,281	50.3
Error	<1–3.4	8,941	0.05	586	0.02	587	0.03
Extra dangling end	9.5–70.1	5,275,752	28.9	744,126	33.8	517,306	33.3
Too close from RES	23–93.7	5,348,693	29.3	642,844	29.2	221,954	14.3
Too short	3.6–20	924,068	5	94,940	4.3	44,972	2.9
Too large	<1–0.14	21	0.0001	1	0.000045	0	0
Over-represented	3.16–13.2	3,362,668	18.4	446,571	20.3	64,224	4.1
Duplicated	1.94–64.2	13,637,482	74.6	1,186,193	53.9	259,406	16.7
Random breaks	<1–3.5	979,909	5.4	129,690	6.3	173,515	11.4
Valid pairs	7.5–98	4,043,904	22.1	848,181	38.5	1,117,826	71.7

**Figure 1:**
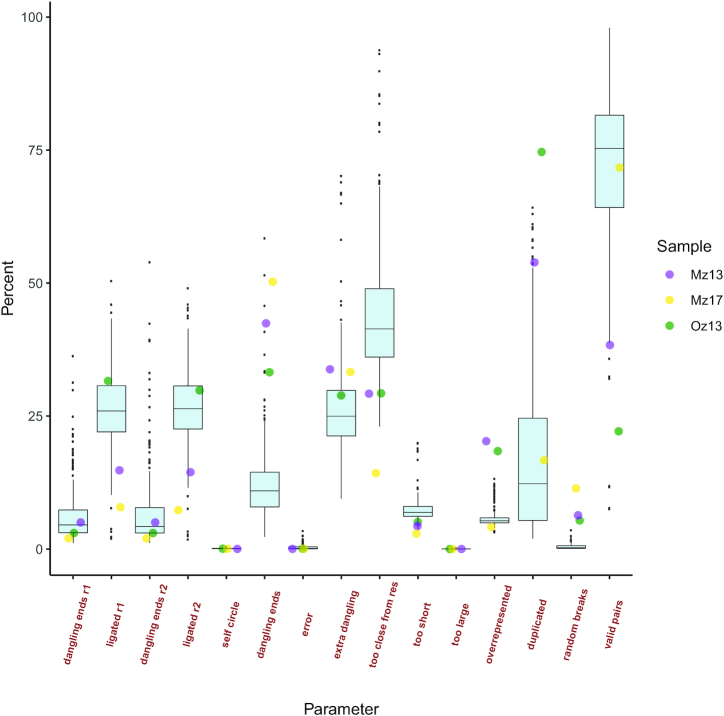
Comparison of values obtained for 15 parameters evaluated in our 3 samples within a context of 316 *in situ* Hi-C samples processed with the same restriction enzyme (RE). From the left, the first 4 parameters are intrinsic values for quality control of the experimental processing before mapping, where “dangling ends r1,2” for each read refers to the number of reads that have been digested but have not been mapped and “ligated” is the number of sites that have been re-ligated (contacting different fragments). The remaining 11 parameters are as follows: Self-circle: both read ends are mapped to the same RE fragment in opposed orientation. Dangling-ends: both read ends are mapped to the same RE fragment in facing orientation. Error: both read ends are mapped to the same RE fragment in the same orientation. Extra dangling end: the read ends are mapped to different RE fragments in facing orientation but are close enough (less than max_molecule_length bp) from the RE cut-site to be considered part of adjacent RE fragments that were not separated by digestion. The max_molecule_length parameter can be inferred from the fragment_size function previously detailed. Too close from RE sites (RES): the start position of 1 of the read ends is too close (5 bp by default) to the RE cutting site. Too short: 1 of the read ends is mapped to RE fragments of <75 bp. These are removed because there is ambiguity on where the read end is mapped because it could also belong to any of the 2 neighbouring RE fragments. Too large: the read ends are mapped to long RE fragments (default: 100 kb, *P* < 10^–5^ to occur in a randomized genome) and they likely represent poorly assembled or repetitive regions. Over-represented: the read ends coming from the top 0.5% most frequently detected RE fragments; they may represent PCR artefacts, random breaks, or genome assembly errors. PCR artefacts or duplicated: the combination of the start positions, mapped length, and strands of both read ends are identical. In this case, only 1 copy is kept. Random break**s**: the start position of 1 read end is too far (less than minimum_distance_to_RE) from the RE cut-site. These are produced most probably by non-canonical enzyme activity or by random physical breakage of the chromatin. Valid pairs: are those pairs of contacting reads that were kept as valid contacts, after removing all other 10 categories of read pairs. Additional details can be found in the filtering function of the TADbit method: https://3dgenomes.github.io/TADbit/tutorial/tutorial_6-Filtering_mapped_reads.html. In the plot, the lower and upper hinges correspond to the first and third quartiles (the 25th and 75th percentiles) and the line in the middle is the median value. The upper whisker extends from the top hinge to the largest value no further than 1.5 * IQR from the hinge (where IQR is the inter-quartile range, or distance between the first and third quartiles). The lower whisker extends from the hinge to the smallest value at most 1.5 * IQR of the hinge. Data beyond the end of the whiskers are called "outlying" points and are plotted individually.

Note that the number of reads does not necessarily add up to the total number of interactions because the same read can be categorized within >1 of the filter categories. Valid pairs represents the number of reads used for generating the Hi-C maps seen in Fig. 1. RES: restriction enzyme sites.

Sample Oz13 yielded the largest number of reads (~29 million), with ~30% of the reads containing ≥1 ligation site, leading to a total of up to ~63% of uniquely mapped reads. The percentage of uniquely mapped reads was much higher for the other 2 samples (up to 78% and 75%, respectively) even though they were sequenced to a much lower depth (Table 1). Using a set of 316 *in situ* Hi-C experiments performed with MboI/DpnII, available at the repository of the 4DGenome unit at the Centre for Genomic Regulation in Barcelona, we placed our 3 BGI processed samples within an Illumina context to compare the obtained values for 15 experimental parameters (Fig. 1).

**Figure 2:**
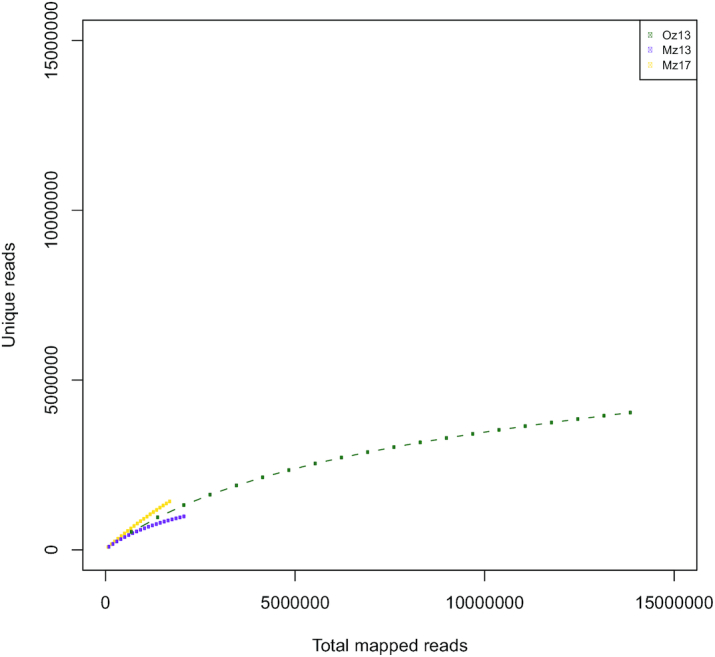
Unique reads per total mapped reads subsampling. Each point within the dotted lines indicates a 5% increase in the total number of the reads (X axis) for each sample. On the Y axis we show the proportion of unique reads mapped for each 5% increment.

The number of reads containing ≥1 ligation site is near optimal and in accordance with what is expected (~30%) for sample Oz13, albeit lower for the other 2 samples (~14% for Mz13 and ~7% for Mz17), but still within the range observed in other Illumina-based Hi-C experiments (Fig. 1). For most of the analysed parameters, the 3 zebra finch samples fall within an expected range, except for duplicates in sample Oz13, which were larger than any of the 316 processed samples, which likely results in the final lower-than-expected number of valid pairs for this sample. We estimated the number of unique reads per total mapped reads (Fig. 2) and found that the high amount of duplicates in sample Oz13 is a consequence of a lack of library complexity as we reached sequencing saturation of the sample (Fig. 2).

We also note that all 3 samples exhibited a slightly (but not critically) higher than expected level of random breaks (Fig. 1), possibly reflecting over-sonication during the library construction process.

The final total number of interactions (the number of read pairs where both read 1 and 2 are mapped) and the uniquely mapped reads can be seen in Table 1 for each sample. Table 2 reports the values represented in Fig. 1, together with the percentage of expected numbers for the 316 samples.

**Figure 3:**
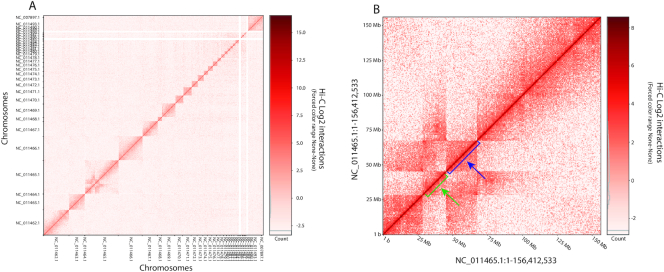
(A) Hi-C Vanilla-normalized contact matrix representation of the genome of sample Oz13. Note the presence of the rearrangement/translocations in chromosome NC_011465.1, as mentioned in the text. (B) Close-up detail of the chromosome NC_011465, Vanilla normalized at a 500-kb resolution. Marked with blue and green arrows are the intrachromosomal rearrangements with relation to the reference genome.

Using TADbit, we then assembled the resulting valid pairs into a 500-kb resolution and Vanilla-normalized [8, 21] the interaction map for sample Oz13 (Fig. 3) and for samples Mz13 and Mz17 (Supplementary Fig. S3). The interaction matrix shows the chromosomal territories for the zebra finch genome, including identification of a translocation/inversion in chromosome NC_011465.1 in comparison to the reference genome (Fig. 3B).

Broadly speaking, standard sequencing experiments usually go beyond the coverage values we present here. Still, in light of our results, deeper sequencing experiments could be performed to obtain more detail in the genome structure. We are confident that the protocol presented here confirms that BGI sequencing protocol generates high-quality reads, potentially suitable for Hi-C pipeline analysis.

## Methods

### *In situ* BGI-Hi-C and sequencing

Samples were processed following the *in situ* BGI-Hi-C protocol, which is described in detail in the Supplementary Materials and Methods. Briefly, the 3 zebra finch tissue samples (50 mg) were coarsely crushed with the help of a scalpel and cross-linked with formaldehyde. Following cross-linking we proceeded to digest DNA with a restriction enzyme (MboI), filling the 5′ overhangs and biotin-tagging the ends of the fragments. We then ligated the resulting blunt-end fragments, sheared the DNA, and retrieved the biotinylated ligated fragments with streptavidin beads. To avoid the risk of DNA loss we skipped the size selection step described in the original protocol and continued with preparing our samples for BGI sequencing. We started by repairing the ends of the DNA fragments and removing the biotin from unligated ends. We then ligated the BGI adapters and filled in the distal overhangs. Finally BGI-Hi-C libraries were indexed in 50 μL volume reactions and amplified for 25 cycles. PCR reaction consisted of 15 μL of BGI-Hi-C library template, 25 μL of 2× Phusion Hi-Fi PCR Master Mix, 0.8 μL bovine serum albumin (20 mg/mL), 2 μL of each primer (10 μM BGI forward primer and indexed reverse primer), 1 μL of dimethyl sulfoxide, and water. Thermocycling conditions were set as follows: 30 s at 98°C, followed by 25 cycles of 30 s at 98°C, 30 s at 60°C, and 30 s at 72°C, and a final 7-min elongation step at 72°C. The number of cycles was estimated using quantitative PCR. Following amplification, PCR products were cleaned using 1× of AmpureXP beads, washing twice with 200 μL of 80% ethanol, followed by a 5-min drying incubation at room temperature. Amplified BGI-Hi-C libraries were eluted in 32 μL of EB buffer after a 5-min incubation at 37°C. BGI-Hi-C libraries were visualized and quantified using a BioAnalyzer instrument and pooled along with other samples. Pooled BGI-Hi-C libraries were circularized, and sequenced as 100PE on the BGISEQ-500 platform at BGI Europe, Copenhagen. Demultiplexing was performed in house, and resulting FastQ files were delivered electronically.

### Data analysis

As a first step of the processing of the sequencing reads, BGI adapters were removed from each sample's FastQ files using cutadapt (v.1.11) [22], with default parameters for PE reads and allowing for 10% mismatch. Trimmed reads were analysed using and following the TADbit [20] pipeline. The TADbit pipeline starts by performing a quality control on the raw data (FastQ files) to assess the quality of the sequencing reads and the efficiency of the digestion and ligation steps of the Hi-C experiments. Next, the PE reads were aligned in TADbit to the available reference genome for the zebra finch (GCF_000151805.1_Taeniopygia_guttata-3.2.4) using the GEM mapper (v2) [23]. Once the reads had been mapped we proceeded to find the intersection of both reads and extract the interacting pairs, followed by a fragment-based filtering step to correct experimental biases/errors. Finally, we binned the valid pairs into a 500-kb resolution and Vanilla-normalized [8, 21] the interaction map.

The detailed bioinformatic pipeline used to analyse the data is available as a Jupyter Notebook and can be found at the project home page.

## Conclusions

As the number of available high-throughput sequencing platforms available increases, each with their own specific profiles of cost, input requirement, and data return, developing the ability to easily tailor experiments to different platforms based on only minor changes to existing protocols is becoming increasingly important.

In this regard, this study represents the first exploration of the applicability of the BGISEQ-500 as an alternative sequencing platform to the Illumina series for the generation of Hi-C sequencing data. With some simple modifications and adaptations to the existing Hi-C protocol, we were able to sequence Hi-C libraries on the BGISEQ-500 platform, proving that there are other options available for researchers who wish to utilize the powerful Hi-C techniques in their research. We find that the modified protocol shows performance similar to that of Illumina experiments. Although we acknowledge that our analyses are limited to a small sample size, our observations suggest that the BGISEQ-500 holds the potential to become a valid and valuable alternative platform for Hi-C data generation that is worthy of future exploration. It is important and it will be interesting for future studies to perform direct comparisons to investigate possible sources of sequencing platform biases, although other studies comparing the BGISEQ and Illumina platforms in other contexts (e.g., exomes, ancient DNA, RNA) show no evidence of such.

## Availability of Supporting Data and Materials

The trimmed sequencing read data supporting the results of this article are available at the University of Copenhagen Electronic Research Data Archive (ERDA repository) [24] with the ID 0af6e87de023ee3508e59a7a868c256b and can be accessed through the archive [24]. Other supporting data are available in the *GigaScience* repository, GigaDB [25].

## Availability of Source Code and Requirements

Project name: BGI-Hi-C Computational Analysis TADbit Pipeline

Project home page: https://github.com/pollicipes/BGI-Hi-C-Computational-Analysis

Operating system(s): Tested for Ubuntu/Linux and MacOSX

Programming language: Python and Bash

Other requirements: Conda and Jupyter Notebook (optional), TADBit and GEM mapper (mandatory)

License: GNU General Public License v3.0

## Additional Files

Supplementary Materials and Methods. BGI-Hi-C protocol

Supplementary Figure S1. BioAnalyzer fragment length distribution images

Supplementary Figure S2. Quality assessment plots of the Hi-C experiment

Supplementary Figure S3. Hi-C contact matrix for samples Mz13 and Mz17

## Abbreviations

bp: base pairs; GC: guanine-cytosine; Hi-C: high-throughput chromosome conformation capture; kb: kilobase pairs; NCBI: National Center for Biotechnology Information; NGS: next-generation sequencing; PE: paired end; IQR: inter-quartile range.

## Competing Interests

The authors declare that they have no competing interests.

## Funding

This work was supported by ERC Consolidator Grant 681,396 “Extinction Genomics” to M.T.P.G. and the Marie-Skłodowska Curie Actions H2020-MSCA-IF-2015, project “EpiCDomestic,” grant number 704,254 to O.S. This research was partially funded by the European Union's Seventh Framework Programme ERC grant agreement 609,989 to M.A.M-R., European Union's Horizon 2020 research and innovation programme grant agreement 676,556 to M.A.M-R. We also acknowledge the support of Spanish Ministerio de Ciencia, Innovación y Universidades through BFU2017–85 926-P to M.A.M-R. and the Generalitat de Catalunya Suport Grups de Recerca AGAUR 2017-SGR-468 to M.A.M-R. C.R.G. acknowledges support from “Centro de Excelencia Severo Ochoa 2013–2017,” SEV-2012–0208 and the CERCA Programme/Generalitat de Catalunya

## Authors' Contributions

M.T.P.G conceived of the study. M.S.-V., O.S., and C.P.E designed the Hi-C experiments and performed the laboratory work. M.S.-V. and O.S. optimized the Hi-C protocol adapted for BGI sequencing. O.S. designed the BGI-specific aspects of the library construction. G.Z. produced the sequencing data. J.A.R. and M.A.M.-R. analysed the data. M.S.-V., O.S., and J.A.R. wrote the manuscript with input from all authors. M.T.P.G., M.A.M.-R., and E.L.A. supervised the work.

## Supplementary Material

giaa087_GIGA-D-20-00080_Original_Submission

giaa087_GIGA-D-20-00080_Revision_1

giaa087_Response_to_Reviewer_Comments_Original_Submission

giaa087_Reviewer_1_Report_Original_Submission Biola M. Javierre -- 4/6/2020 Reviewed

giaa087_Reviewer_1_Report_Revision_1 Biola M. Javierre -- 6/17/2020 Reviewed

giaa087_Reviewer_2_Report_Original_Submission Hyun Park, Ph.D -- 4/20/2020 Reviewed

giaa087_Reviewer_3_Report_Original_Submission Ellie Armstrong -- 4/30/2020 Reviewed

giaa087_Supplemental_File

## References

[1] Cullen KE , Kladde MP, Seyfred MA. Interaction between transcription regulatory regions of prolactin chromatin. Science. 1993;261:203–6. 8327891 10.1126/science.8327891

[2] Dekker J , Rippe K, Dekker M, et al. Capturing chromosome conformation. Science. 2002;295:1306–11. 11847345 10.1126/science.1067799

[3] Grob S , Cavalli G. Technical review: A hitchhiker's guide to chromosome conformation capture. In: Bemer M, Baroux Ceds. Plant Chromatin Dynamics. New York, NY: Humana; 2018:233–46. 10.1007/978-1-4939-7318-7_14 29052195

[4] Lieberman-Aiden E , van Berkum NL, Williams L, et al. Comprehensive mapping of long-range interactions reveals folding principles of the human genome. Science. 2009;326:289–93. 19815776 10.1126/science.1181369 PMC2858594

[5] Simonis M , Klous P, Splinter E, et al. Nuclear organization of active and inactive chromatin domains uncovered by chromosome conformation capture–on-chip (4C). Nat Genet. 2006;38:1348–54. 17033623 10.1038/ng1896

[6] Dostie J , Richmond TA, Arnaout RA, et al. Chromosome Conformation Capture Carbon Copy (5C): a massively parallel solution for mapping interactions between genomic elements. Genome Res. 2006;16:1299–309. 16954542 10.1101/gr.5571506 PMC1581439

[7] Ferraiuolo MA , Sanyal A, Naumova N, et al. From cells to chromatin: Capturing snapshots of genome organization with 5C technology. Methods. 2012;58:255–67. 23137922 10.1016/j.ymeth.2012.10.011 PMC3874844

[8] Rao SSP , Huntley MH, Durand NC, et al. A 3D map of the human genome at kilobase resolution reveals principles of chromatin looping. Cell. 2014;159:1665–80. 25497547 10.1016/j.cell.2014.11.021 PMC5635824

[9] Schoenfelder S , Furlan-Magaril M, Mifsud B, et al. The pluripotent regulatory circuitry connecting promoters to their long-range interacting elements. Genome Res. 2015;25:582–97. 25752748 10.1101/gr.185272.114 PMC4381529

[10] Schoenfelder S , Javierre B-M, Furlan-Magaril M, et al. Promoter capture Hi-C: High-resolution, genome-wide profiling of promoter interactions. J Vis Exp. 2018, doi:10.3791/57320. PMC6102006 30010637

[11] Belyaeva A , Venkatachalapathy S, Nagarajan M, et al. Network analysis identifies chromosome intermingling regions as regulatory hotspots for transcription. Proc Natl Acad Sci U S A. 2017;114:13714–9. 29229825 10.1073/pnas.1708028115 PMC5748172

[12] Friedman N , Rando OJ. Epigenomics and the structure of the living genome. Genome Res. 2015;25:1482–90. 26430158 10.1101/gr.190165.115 PMC4579333

[13] Fraser J , Williamson I, Bickmore WA. An overview of genome organization and how we got there: from FISH to Hi-C. Microbiol Mol Biol Rev. 2015;79:347–72. 26223848 10.1128/MMBR.00006-15 PMC4517094

[14] Drmanac R , Sparks AB, Callow MJ, et al. Human genome sequencing using unchained base reads on self-assembling DNA nanoarrays. Science. 2010;327:78–81. 19892942 10.1126/science.1181498

[15] Goodwin S , McPherson JD, McCombie WR. Coming of age: Ten years of next-generation sequencing technologies. Nat Rev Genet. 2016;17:333–51. 27184599 10.1038/nrg.2016.49 PMC10373632

[16] Mak SST , Gopalakrishnan S, Carøe C, et al. Comparative performance of the BGISEQ-500 vs Illumina HiSeq2500 sequencing platforms for palaeogenomic sequencing. Gigascience. 2017;6, doi:10.1093/gigascience/gix049. PMC5570000 28854615

[17] Smith O , Dunshea G, Sinding M-HS, et al. Ancient RNA from Late Pleistocene permafrost and historical canids shows tissue-specific transcriptome survival. PLoS Biol. 2019;17:e3000166. 31361744 10.1371/journal.pbio.3000166 PMC6667121

[18] Xu Y , Lin Z, Tang C, et al. A new massively parallel nanoball sequencing platform for whole exome research. BMC Bioinformatics. 2019;20:153. 30909888 10.1186/s12859-019-2751-3 PMC6434795

[19] Zhu F-Y , Chen M-X, Ye N-H, et al. Comparative performance of the BGISEQ-500 and Illumina HiSeq4000 sequencing platforms for transcriptome analysis in plants. Plant Methods. 2018;14:69. 30123314 10.1186/s13007-018-0337-0 PMC6088413

[20] Serra F , Baù D, Goodstadt M, et al. Automatic analysis and 3D-modelling of Hi-C data using TADbit reveals structural features of the fly chromatin colors. PLoS Comput Biol. 2017;13:e1005665. 28723903 10.1371/journal.pcbi.1005665 PMC5540598

[21] Imakaev M , Fudenberg G, McCord RP, et al. Iterative correction of Hi-C data reveals hallmarks of chromosome organization. Nat Methods. 2012;9:999–1003. 22941365 10.1038/nmeth.2148 PMC3816492

[22] Martin M . Cutadapt removes adapter sequences from high-throughput sequencing reads. EMBnet J. 2011;17:10–2.

[23] Marco-Sola S , Sammeth M, Guigó R, et al. The GEM mapper: Fast, accurate and versatile alignment by filtration. Nat Methods. 2012;9:1185–8. 23103880 10.1038/nmeth.2221

[24] Copenhagen Electronic Research Data Archive . https://sid.erda.dk/public/archives/0af6e87de023ee3508e59a7a868c256b/published-archive.html. Accessed 10 July 2020.

[25] Sandoval-Velasco M , Rodríguez JA, Perez Estrada C, et al. Supporting data for “Hi-C chromosome conformation capture sequencing of avian genomes using the BGISEQ-500 platform.”. GigaScience Database. 2020. 10.5524/100770. PMC7448675 32845983

