# Supplementary material for: Hi-C chromosome conformation capture sequencing of avian genomes using the BGISEQ-500 platform
Source: Gigascience. 2020 Aug 26;9(8):giaa087. doi: 10.1093/gigascience/giaa087 (PMC7448675; doi:10.1093/gigascience/giaa087)
Supplement: giaa087_GIGA-D-20-00080_Original_Submission [file giaa087_giga-d-20-00080_original_submission.pdf]

GigaScience

Hi-C chromosome conformation capture sequencing of avian genomes using the BGISEQ-500 platform

--Manuscript Draft--

**Manuscript Number:**	GIGA-D-20-00080
**Full Title:**	Hi-C chromosome conformation capture sequencing of avian genomes using the BGISEQ-500 platform
**Article Type:**	Technical Note
**Funding Information:**	H2020 European Research Council (Consolidator) (681396) — Prof. M. Thomas P. Gilbert<br>H2020 Marie Skłodowska-Curie Actions (IF) (704254) — Dr. Oliver Smith<br>Seventh Framework Programme (ERC) (609989) — Prof. Marc A. Marti-Renom<br>Horizon 2020 Research and Innovation Programme (676556) — Prof. Marc A. Marti-Renom<br>Ministerio de Ciencia, Innovación y Universidades (BFU2017-85926-P) — Prof. Marc A. Marti-Renom<br>Generalitat de Catalunya Suport Grups de Recerca (AGAUR 2017-SGR-468) — Prof. Marc A. Marti-Renom
**Abstract:**	Background<br>Hi-C experiments couple DNA-DNA proximity with next-generation sequencing to yield an unbiased description of genome wide interactions. Previous methods describing Hi-C experiments have focused on the industry-standard Illumina sequencing. With new next-generation sequencing platforms such as BGISEQ-500 becoming more widely available, protocol adaptations to fit platform- specific requirements are useful to give increased choice to researchers who routinely generate sequencing data.<br>Results<br>We describe an in-situ Hi-C protocol adapted to be compatible with the the BGISEQ-500 high-throughput sequencing platform. Using zebra finch (Taeniopygia guttata) as a biological sample, we demonstrate how Hi-C libraries can be constructed to generate informative data using the BGISEQ-500 platform, following circularisation and DNA nanoball generation. Our protocol is a modification of an Illumina-compatible method, based around blunt-end ligations in library construction, using un- barcoded, distally overhanging double-stranded adapters, followed by amplification using indexed primers. This resulting libraries are ready for circularisation and subsequent sequencing on the BGISEQ series of platforms, and yield data similar to what can be expected using Illumina-compatible approaches.<br>Conclusions<br>Our straightforward modification to an Illumina-compatible in-situ Hi-C protocol enables data generation on the BGISEQ series of platforms, thus expanding the options available for researchers who wish to utilise the powerful Hi-C techniques in their research.
**Corresponding Author:**	Marcela Sandoval-Velasco<br>University of Copenhagen<br>Copenhagen, DENMARK
**Corresponding Author Secondary Information:**	
**Corresponding Author's Institution:**	University of Copenhagen
**Corresponding Author's Secondary Institution:**	
**First Author:**	Marcela Sandoval-Velasco

Powered by Editorial Manager® and ProduXion Manager® from Aries Systems Corporation

First Author Secondary Information:	
Order of Authors:	Marcela Sandoval-Velasco
	Juan Antonio Rodríguez
	Cynthia Perez Estrada
	Guojie Zhang
	Erez Lieberman Aiden
	Marc A. Marti-Renom
	M. Thomas P. Gilbert
	Oliver Smith
Order of Authors Secondary Information:	
Additional Information:	
Question	Response
Are you submitting this manuscript to a special series or article collection?	No
**Experimental design and statistics**<br><br>Full details of the experimental design and statistical methods used should be given in the Methods section, as detailed in our Minimum Standards Reporting Checklist. Information essential to interpreting the data presented should be made available in the figure legends.<br><br>Have you included all the information requested in your manuscript?	Yes
**Resources**<br><br>A description of all resources used, including antibodies, cell lines, animals and software tools, with enough information to allow them to be uniquely identified, should be included in the Methods section. Authors are strongly encouraged to cite Research Resource Identifiers (RRIDs) for antibodies, model organisms and tools, where possible.<br><br>Have you included the information requested as detailed in our Minimum Standards Reporting Checklist?	Yes

Powered by Editorial Manager® and ProduXion Manager® from Aries Systems Corporation

**Availability of data and materials**<br><br>All datasets and code on which the conclusions of the paper rely must be either included in your submission or deposited in publicly available repositories (where available and ethically appropriate), referencing such data using a unique identifier in the references and in the “Availability of Data and Materials” section of your manuscript.<br><br>Have you have met the above requirement as detailed in our Minimum Standards Reporting Checklist?	Yes

Manuscript with figures and tables
Click here to download Manuscript SandovalVelasco_etal_GigascienceTN_Main.pdf

Hi-C chromosome conformation capture sequencing of avian genomes using the BGISEQ-500 platform

Marcela Sandoval-Velasco[1*], Juan Antonio Rodríguez[2*], Cynthia Perez Estrada[3], Guojie Zhang[4], Erez Lieberman Aiden[3,5,6,7], Marc A. Marti Renom[2,8,9,10], M. Thomas P. Gilbert[1,11], & Oliver Smith[1,12]

[1] Section for Evolutionary Genomics, University of Copenhagen, 1353 Copenhagen, Denmark
[2] CNAG-CRG, Centre for Genomic Regulation, Barcelona Institute of Science and Technology, 08028 Barcelona, Spain
[3] Center for Genome Architecture, Department of Molecular and Human Genetics, Baylor College of Medicine, Houston TX, USA
[4] China National GeneBank, BGI-Shenzhen, Shenzhen 518083, China
[5] Center for Theoretical Biological Physics, Rice University, Houston TX, USA
[6] Broad Institute of the Massachusetts Institute of Technology and Harvard University, Cambridge MA, USA
[7] Department of Computer Science and Computational Applied Mathematics, Rice University, Houston TX, USA
[8] Centre for Genomic Regulation, The Barcelona Institute for Science and Technology, Carrer del Doctor Aiguader 88, Barcelona, 08003, Spain
[9] Pompeu Fabra University, Doctor Aiguader 88, Barcelona, 08003, Spain
[10] ICREA, Pg. Lluís Companys 23, 08010 Barcelona, Spain
[11] Norwegian University of Science and Technology, University Museum, 7491 Trondheim, Norway
[12] Micropathology Ltd, University of Warwick Science Park, Coventry CV4 7EZ, UK

*M.S.-V and J.A.R contributed to this work equally

Corresponding authors:
marcela.velasco@sund.ku.dk
tgilbert@sund.ku.dk
oliver.smith@palaeome.org

For GiGaScience as a Technical Note

Abstract

Background

Hi-C experiments couple DNA-DNA proximity with next-generation sequencing to yield an unbiased description of genome wide interactions. Previous methods describing Hi-C experiments have focused on the industry-standard Illumina sequencing. With new next-generation sequencing platforms such as BGISEQ-500 becoming more widely available, protocol adaptations to fit platform-specific requirements are useful to give increased choice to researchers who routinely generate sequencing data.

Results

We describe an in-situ Hi-C protocol adapted to be compatible with the the BGISEQ-500 high-throughput sequencing platform. Using zebra finch (*Taeniopygia guttata*) as a biological sample, we demonstrate how Hi-C libraries can be constructed to generate informative data using the BGISEQ-500 platform, following circularisation and DNA nanoball generation. Our protocol is a modification of an Illumina-compatible method, based around blunt-end ligations in library construction, using un-barcoded, distally overhanging double-stranded adapters, followed by amplification using indexed primers. This resulting libraries are ready for circularisation and subsequent sequencing on the BGISEQ series of platforms, and yield data similar to what can be expected using Illumina-compatible approaches.

Conclusions

Our straightforward modification to an Illumina-compatible in-situ Hi-C protocol enables data generation on the BGISEQ series of platforms, thus expanding the options available for researchers who wish to utilise the powerful Hi-C techniques in their research.

Keywords

Hi-C, BGISEQ-500, Next Generation Sequencing, chromosome conformation capture

Background

Determining the organisation of chromatin and chromosomes within a cell nucleus carries great potential for describing genome function, regulation, interactions, and evolution. Advancements in molecular techniques since the beginning of the 21st century have allowed steady progress in uncovering these interactions using various forms of DNA-DNA proximity ligation [1] and Chromosome Conformation Capture (3C) techniques [2]. These methods involve the covalent crosslinking of chromatin regions interacting in space, followed by restriction enzyme digestion, and proximity ligation of fragments from interacting regions. This is then followed by DNA sequencing, yielding data that can be used to describe proximal interactions between distant genomic loci [3]. Earlier versions known as 3C and 4C generally focused on single or few loci, using locus-specific primers and sanger sequencing [4,5]. Later, multiple parallel loci contacts could be quantified at once using either quantitative PCR approaches, microarray characterisation, or early next-generation sequencing (NGS) platforms such as Roche 454, in a protocol known as 5C [6,7].

More recently however, NGS methods coupled with tailored bioinformatic tools have allowed a full range of interactions to be described at the genome scale using a method known as Hi-C [4]. These advances allow for insights not only into overall chromatin structure [8], but into the interactions between genes and their regulatory elements [9,10] and their functions [11], revealing further layers of epigenomic activity to be decoded [12]. These genome-scale Hi-C analyses have used established NGS platforms as the preferred sequencing method, particularly those with short-read characteristics. Indeed, pair-end sequencing offered by the Illumina platforms [13] is widely used in Hi-C due to their ubiquity and relatively universal final library constructs. In Hi-C, the mapping of both ends of a single read reveals a high sequential distance but at the same time implies a close physical proximity of the two ends, allowing for the identification of chromosomal contacts. While long-read platforms such as PacBio are invaluable for *de novo* sequencing approaches due to their ability to overcome nebulous genomic regions such as short tandem repeats or AT-rich regions, they cannot provide details of the structure of a genome. Therefore, for the time being, short, paired-end reads is the sequencing method of choice for identifying spatially close loci using 3C-based methods.

Although Illumina platforms have dominated the sequencing for these type of methods, alternative technologies are now appearing. One of these is the BGISEQ platform whose general workflow and stepwise sequencing procedures are similar to those of Illumina series, yet the sequencing templates have marked differences. Illumina employs hybridisation clustering followed by bridge amplification [13], whereas in the BGISEQ, the DNA undergoes an iterative ligation, circularization and amplification process that generates billions of DNA nanoballs that are then deposited on a flow cell [14]. Comparisons between the Illumina HiSeq-2500 and BGISEQ-500 platforms have been made previously for the sequencing of shotgun DNA [15] and RNA [16], with both studies finding very little difference between platforms for standard metrics for each molecule type such as clonality, endogenous content, GC content, and sequence quality scores. Furthermore, the Illumina HiSeq-4000 and BGISEQ-500 have also been compared for sequencing of exomes [17] and transcriptomes [18], validating the capability of BGISEQ-500 to be established as a competitive and reliable platform for both exome and transcriptome analysis.

Given that the performance of the BGISEQ-500 as a sequencer is similar to Illumina, we explored whether the Hi-C method could be adapted to the BGISEQ-500 platform. Specifically we adapted the in-situ Hi-C protocol published by Rao *et al* [5] by changing the adapter ligation step to employ blunt-end ligation, meaning omission of the A-tailing step in Illumina library construction. Additionally, we introduced a post-ligation fill-in step to remove overhangs present on the distal ends of the BGISEQ adapters. We also modified the sequences of the adapters and amplification primers to be compatible with the BGISEQ-500 platform. We then submitted amplified libraries to BGI-Europe for circularisation, DNA nanoball construction, and sequencing, and analysed the data to assess the method's performance.

Results

We processed 3 different zebra finch (*Taeniopygia guttata*) tissue samples using the BGI-Hi-C protocol developed for this study and described in detail in the Supplementary Information. The BGI-Hi-C libraries were quantified and visualized using a BioAnalyzer Instrument (Fig. S.1), pooled and sequenced on a partial lane of the BGISEQ-500 with PE100 sequencing mode. Sequencing reads were analyzed using TADbit [19], a pipeline developed for Hi-C experiments to pre-process the reads, assess the quality of the Hi-C experiments (Fig. S.2), map the reads to a reference genome, filter and normalize interaction data, analyze the resulting interaction matrices, and generate statistics and maps to model and explore 3C-based data.

The three zebra finch samples yielded approximately 29, 3 and 2 million paired-end sequence reads, respectively (Table 1). Using the TADbit pipeline we mapped and filtered all reads for the three samples (Table 2). Mapping was performed following a fragment based method implemented in TADbit [19]. To map the sequence data we used the available reference genome on NCBI for the Zebra finch (GCF_000151805.1_Taeniopygia_guttata-3.2.4).

Sample Oz13 yielded the largest number of reads (~29M), with approximately 30% of the reads containing at least one ligation site, leading to a total of up to ~63% of uniquely mapped reads. The percentage of uniquely mapped reads was much higher for the other two samples (up to 78% and 75%, respectively) even though they were sequenced to a much lower depth (Table 1). Using a set of 320 *in situ* Hi-C experiments performed with MboI/DpnII, available at the repository of the 4DGenome unit at the Centre for Genomic Regulation in Barcelona, we placed our three BGI processed samples within an Illumina context to compare the obtained values for 15 experimental parameters (Fig. 1).

The number of reads containing at least one ligation site is near optimal and in accordance to what is expected (~30%) for sample Oz13, albeit lower for the other two samples (~14% for Mz13 and ~7% for Mz17), but still within the range observed in other Illumina based Hi-C experiments (Fig. 1). For most of the analyzed parameters, the 3 zebra finch samples fall within an expected range, except for duplicates in sample Oz13, which were larger than any of the 320 processed samples, which likely results in the final lower than expected valid pairs for this sample. We estimated the number of unique reads per total mapped reads (Fig. 2), and found that the high amount of duplicates in sample Oz13 is a consequence of a lack of library complexity as we reached sequencing saturation of the sample (Fig. 2).

We also note that all three samples exhibited a slightly (but not critically) higher than expected level of random breaks (Fig. 1), possibly reflecting over sonication during the library construction process.

The final total number of interactions (the number of read pairs where both read 1 and 2 are mapped) and the uniquely mapped reads can be seen at Table 1 for each sample. Table 2 shows the values represented in Figure 1, together with the percentage of expected numbers for the 320 samples.

Using TADbit, we then assembled the resulting valid-pairs into a 500 kb resolution and Vanilla-normalized [8,20] the interaction map for sample Oz13 (Fig. 3) and for samples Mz13 and Mz17 (Fig. S.3). The interaction matrix shows the chromosomal territories for the zebra finch genome, including identification of a translocation/inversion in chromosome NC_011465.1 in comparison to the reference genome (Fig. 3B).

Broadly speaking, standard sequencing experiments usually go beyond the coverage values we present in here. Still, in light of our results, deeper sequencing experiments could be performed to

obtain more in detail in the genome structure. We are confident that the protocol presented here confirms that BGI sequencing protocol generates good quality reads, potentially suitable for Hi-C pipeline analysis.

Methods description

In-situ BGI-Hi-C and sequencing

Samples were processed following the in-situ BGI-Hi-C protocol that can be found described in detail in the Supplementary Materials and Methods. Briefly, the three zebra finch tissue samples were coarsely crushed with the help of a scalpel and crosslinked with formaldehyde. Following crosslinking we proceeded to digest DNA with a restriction enzyme (MboI), filling the 5' overhangs and biotin-tagging the ends of the fragments. We then ligated the resulting blunt-end fragments, sheared the DNA, and retrieved the biotinylated ligated fragments with streptavidin beads. We skipped the size selection step described in the protocol, and continued with preparing our samples for BGI sequencing. We started by repairing the ends of the DNA fragments and removing the biotin from unligated ends. We then ligated the BGI adapters and filled-in the distal overhangs. Finally BGI-Hi-C libraries were indexed in 50 µl volume reactions and amplified for 25 cycles. PCR reaction consisted of 15 µl of BGI-Hi-C library template, 25 µl of 2x Phusion Hi-Fi PCR Master Mix, 0.8 µl BSA (20 mg/ml), 2 µl of each primer (10 µM, BGI forward primer and indexed reverse primer), 1 µl of DMSO and water. Thermocycling conditions were set to: 30 sec at 98°C, followed by 25 cycles of 30s at 98°C, 30s at 60°C and 30s at 72°C, and a final 7 min elongation step at 72°C. The number of cycles was estimated using qPCR. Following amplification, PCR products were cleaned using 1X of AmpureXP beads, washing twice with 200µL of 80% EtOH, followed by a 5 min drying incubation at RT. Amplified BGI-Hi-C libraries were eluted in 32µL of EB buffer after a 5 min incubation at 37ºC. BGI-Hi-C libraries were visualized and quantified using a BioAnalyzer instrument, and pooled along with other samples. Pooled BGI-Hi-C libraries were circularised, and sequenced as 100PE on the BGISEQ-500 platform at BGI Europe, Copenhagen. Demultiplexing was performed in-house, and resulting FastQ files were delivered electronically.

Data analysis

As a first step of the processing of the sequencing reads, BGI adapters were removed from each sample FastQ files using cutadapt (v.1.11) [21], with default parameters for pair-end reads and allowing for 10% mismatch. Trimmed reads were analyzed using and following the TADbit [19] pipeline. The TADbit pipeline starts by performing a quality control on the raw data (FastQ files) to assess the quality of the sequencing reads and the efficiency of the digestion and ligation steps of the Hi-C experiments. Next, the paired-end reads were aligned in TADbit to the available reference genome for the Zebra finch (GCF_000151805.1_Taeniopygia_guttata-3.2.4) using the GEM mapper (v2) [22]. Once the reads had been mapped we proceeded to find the intersection of both reads and extract the interacting pairs, followed by a fragment-based filtering step to correct experimental biases/errors. Finally, we binned the valid-pairs into a 500 kb resolution and Vanilla-normalized [8,20] the interaction map.
The detailed bioinformatic pipeline used to analyze the data is available as a Jupyter Notebook and can be found at https://github.com/pollicipes/BGI-HiC-Computational-Analysis.

Conclusions

As the number of available high-throughput sequencing platforms available increases, each with their own specific profiles of cost, input requirement, and data return, developing the ability to easily tailor

experiments to different platforms based on only minor changes to existing protocols is becoming increasingly important.

In this regard, this study represents the first exploration of the applicability of the BGISEQ-500 as an alternative sequencing platform to the Illumina series for the generation of Hi-C sequencing data. With some simple modifications and adaptations to the existing Hi-C protocol, we were able to sequence Hi-C libraries on the BGISEQ-500 platform, proving that there are other options available for researchers who wish to utilise the powerful Hi-C techniques in their research. We find that the modified protocol shows similar performance to Illumina experiments. Although we acknowledge that our analyses are limited to a small sample size, our observations suggest that the BGISEQ-500 holds the potential to a valid and valuable alternative platform for Hi-C data generation that is worthy of future exploration. It is important and it will be interesting for future studies to do direct comparisons to investigate possible sources of sequencing platform biases, although other studies comparing the BGISEQ and Illumina platforms in other contexts (e.g. exomes, ancient DNA, RNA) show no evidence of such.

Availability of Supporting data

The trimmed sequencing read data supporting the results of this article is available at the University of Copenhagen Electronic Research Data Archive (ERDA repository) and can be accessed through: https://sid.erda.dk/public/archives/0af6e87de023ee3508e59a7a868c256b/published-archive.html

Availability of Source Code and Requirements

Project name: BGI-HiC Computational Analysis TADbit Pipeline
Project home page: https://github.com/pollicipes/BGI-HiC-Computational-Analysis
Operating system(s): Tested for Ubuntu/Linux and MacOSX
Programming language: Python and Bash
Other requirements: Conda & Jupyter Notebook (optional), TADBit & GEM mapper (mandatory)
License: GNU General Public License v3.0

Abreviations

Hi-C: high-throughput chromosome conformation capture; NGS: Next Generation Sequencing; PE: paired-end; PCR: Polymerase Chain Reaction; qPCR: quantitative Polymerase Chain Reaction; RT: Room Temperature; NCBI: National Center for Biotechnology Information.

Competing interests

The authors declare no conflicts of interest.

Funding

This work was supported by ERC Consolidator Grant 681396 ‘Extinction Genomics’ to MTPG and the Marie-Skłodowska Curie Actions H2020-MSCA-IF-2015, project ‘EpiCDomestic’, grant number 704254 to OS. This research was partially funded by the European Union’s Seventh Framework Programme the ERC grant agreement 609989 to M.A.M-R., European Union’s Horizon 2020 research

and innovation programme grant agreement 676556 to M.A.M-R. We also acknowledge the support of Spanish Ministerio de Ciencia, Innovación y Universidades through BFU2017-85926-P to M.A.M-R. and the Generalitat de Catalunya Suport Grups de Recerca AGAUR 2017-SGR-468 to M.A.M-R. CRG acknowledges support from 'Centro de Excelencia Severo Ochoa 2013-2017', SEV-2012-0208 and the CERCA Programme/Generalitat de Catalunya.

Authors' contributions

M.T.P.G conceived of the study. M.S.-V, O.S and C.P.E designed the Hi-C experiments and performed the lab work. M.S.-V and O.S optimised the Hi-C protocol adapted for BGI sequencing. O.S designed the BGI-specific aspects of the library construction. G.Z. produced the sequencing data. J.A.R and M.A.M.-R analyzed the data. M.S.-V, O.S and J.A.R wrote the manuscript with input from all the authors. M.T.P.G, M.A.M.-R and E.L.A supervised the work.

Acknowledgements

The authors would like to thank and acknowledge the Vertebrate Genome Laboratory at The Rockefeller University for providing the tissue samples under study, and the BGI-EUROPE Sequencing facility for help and assistance during the BGISEQ data generation.

References

1. Cullen KE, Kladde MP, Seyfred MA. Interaction between transcription regulatory regions of prolactin chromatin. Science. 1993;261:203–6.

2. Dekker J, Rippe K, Dekker M, Kleckner N. Capturing chromosome conformation. Science. 2002;295:1306–11.

3. Grob S, Cavalli G. Technical Review: A Hitchhiker's Guide to Chromosome Conformation Capture [Internet]. Methods in Molecular Biology. 2018. p. 233–46. Available from: http://dx.doi.org/10.1007/978-1-4939-7318-7_14

4. Lieberman-Aiden E, van Berkum NL, Williams L, Imakaev M, Ragoczy T, Telling A, et al. Comprehensive mapping of long-range interactions reveals folding principles of the human genome. Science. 2009;326:289–93.

5. Simonis M, Klous P, Splinter E, Moshkin Y, Willemsen R, de Wit E, et al. Nuclear organization of active and inactive chromatin domains uncovered by chromosome conformation capture–on-chip (4C). Nat Genet. 2006;38:1348–54.

6. Dostie J, Richmond TA, Arnaout RA, Selzer RR, Lee WL, Honan TA, et al. Chromosome Conformation Capture Carbon Copy (5C): a massively parallel solution for mapping interactions between genomic elements. Genome Res. 2006;16:1299–309.

7. Ferraiuolo MA, Sanyal A, Naumova N, Dekker J, Dostie J. From cells to chromatin: capturing snapshots of genome organization with 5C technology. Methods. 2012;58:255–67.

8. Rao SSP, Huntley MH, Durand NC, Stamenova EK, Bochkov ID, Robinson JT, et al. A 3D map of the human genome at kilobase resolution reveals principles of chromatin looping. Cell. 2014;159:1665–80.

9. Schoenfelder S, Furlan-Magaril M, Mifsud B, Tavares-Cadete F, Sugar R, Javierre B-M, et al. The pluripotent regulatory circuitry connecting promoters to their long-range interacting elements. Genome

Res. 2015;25:582–97.

10. Schoenfelder S, Javierre B-M, Furlan-Magaril M, Wingett SW, Fraser P. Promoter Capture Hi-C: High-resolution, Genome-wide Profiling of Promoter Interactions [Internet]. Journal of Visualized Experiments. 2018. Available from: http://dx.doi.org/10.3791/57320

11. Belyaeva A, Venkatachalapathy S, Nagarajan M, Shivashankar GV, Uhler C. Network analysis identifies chromosome intermingling regions as regulatory hotspots for transcription. Proc Natl Acad Sci U S A. 2017;114:13714–9.

12. Friedman N, Rando OJ. Epigenomics and the structure of the living genome. Genome Res. 2015;25:1482–90.

13. Fraser J, Williamson I, Bickmore WA. An overview of genome organization and how we got there: from FISH to Hi-C. Microbiol Mol Biol Rev [Internet]. Am Soc Microbiol; 2015; Available from: https://mmbr.asm.org/content/79/3/347.short

14. Goodwin S, McPherson JD, McCombie WR. Coming of age: ten years of next-generation sequencing technologies. Nat Rev Genet. 2016;17:333–51.

15. Mak SST, Gopalakrishnan S, Carøe C, Geng C, Liu S, Sinding M-HS, et al. Comparative performance of the BGISEQ-500 vs Illumina HiSeq2500 sequencing platforms for palaeogenomic sequencing. Gigascience. 2017;6:1–13.

16. Smith O, Dunshea G, Sinding M-HS, Fedorov S, Germonpre M, Bocherens H, et al. Ancient RNA from Late Pleistocene permafrost and historical canids shows tissue-specific transcriptome survival. PLoS Biol. 2019;17:e3000166.

17. Xu Y, Lin Z, Tang C, Tang Y, Cai Y, Zhong H, et al. A new massively parallel nanoball sequencing platform for whole exome research. BMC Bioinformatics. 2019;20:153.

18. Zhu F-Y, Chen M-X, Ye N-H, Qiao W-M, Gao B, Law W-K, et al. Comparative performance of the BGISEQ-500 and Illumina HiSeq4000 sequencing platforms for transcriptome analysis in plants. Plant Methods. 2018;14:69.

19. Serra F, Baù D, Goodstadt M, Castillo D, Filion GJ, Marti-Renom MA. Automatic analysis and 3D-modelling of Hi-C data using TADbit reveals structural features of the fly chromatin colors. PLoS Comput Biol. 2017;13:e1005665.

20. Imakaev M, Fudenberg G, McCord RP, Naumova N, Goloborodko A, Lajoie BR, et al. Iterative correction of Hi-C data reveals hallmarks of chromosome organization. Nat Methods. 2012;9:999–1003.

21. Martin M. Cutadapt removes adapter sequences from high-throughput sequencing reads. EMBnet.journal. 2011;17:10–2.

22. Marco-Sola S, Sammeth M, Guigó R, Ribeca P. The GEM mapper: fast, accurate and versatile alignment by filtration. Nat Methods. 2012;9:1185–8.

Figures and Tables

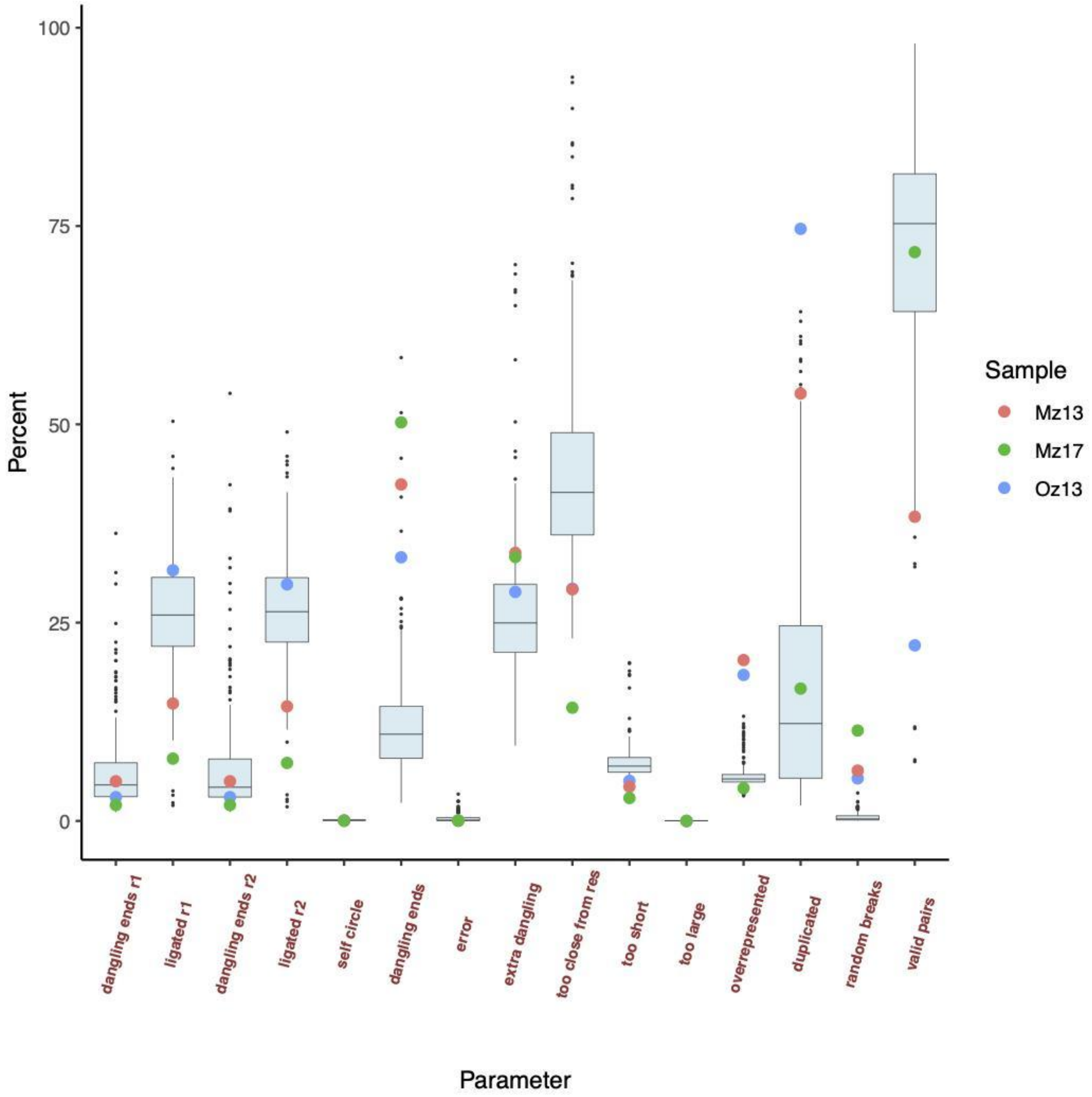

Figure 1: Comparison of values obtained for 15 parameters evaluated in our 3 samples within a context of 320 *in-situ* Hi-C samples processed with the same restriction enzyme (RE). From the left, first 4 parameters are intrinsic values for quality control of the experimental processing before mapping; where "*dangling ends r1,2*" for each read refers to the number of reads that have been digested, but have not been mapped, and "*ligated*" is the number of sites that have been re-ligated (contacting different fragments). The remaining 11 parameters are: **Self-circle**: both read-ends are mapped to the same RE fragment in *opposed* orientation. **Dangling-end**: both read-ends are mapped to the same RE fragment in *facing* orientation. **Error**: both read-ends are mapped to the same RE fragment in the same orientation. **Extra dangling-end**: the read-ends are mapped to different RE fragments in *facing* orientation, but are close enough (< *max_molecule_length* bp) from the RE cut-site to be considered part of adjacent RE fragments that were not separated by digestion. The *max_molecule_length* parameter can be inferred from the *fragment_size* function previously detailed. **Too close from RE sites**: the start position of one of the read-end is too close (5 bp by default) from the RE cutting site. **Too short**: one of the read-ends is mapped to RE fragments of less than 75bp. These are removed since there is ambiguity on where the read-end is mapped as it could also belong to any of the two neighboring RE fragments. **Too large**: the read-ends are mapped to long RE fragments (default: 100 kb, P < 10-5 to occur in a randomized genome) and they likely represent poorly assembled or repetitive regions. **Over-represented**: the read-ends coming from the top 0.5% most frequently detected RE fragments, they may represent PCR artefacts, random breaks, or genome assembly errors. **PCR artefacts or duplicated**: the combination of the start positions, mapped length, and strands of both read-ends are identical. In this case, only one copy is kept. **Random breaks**: the start position of one read-end is too far (> *minimum_distance_to_RE*) from the RE cut-site. These are produced most probably by non-canonical enzyme activity or by random physical breakage of the chromatin. Additional details can be found in the filtering function of the TADbit method: https://3dgenomes.github.io/TADbit/tutorial/tutorial_6-Filtering_mapped_reads.html

-

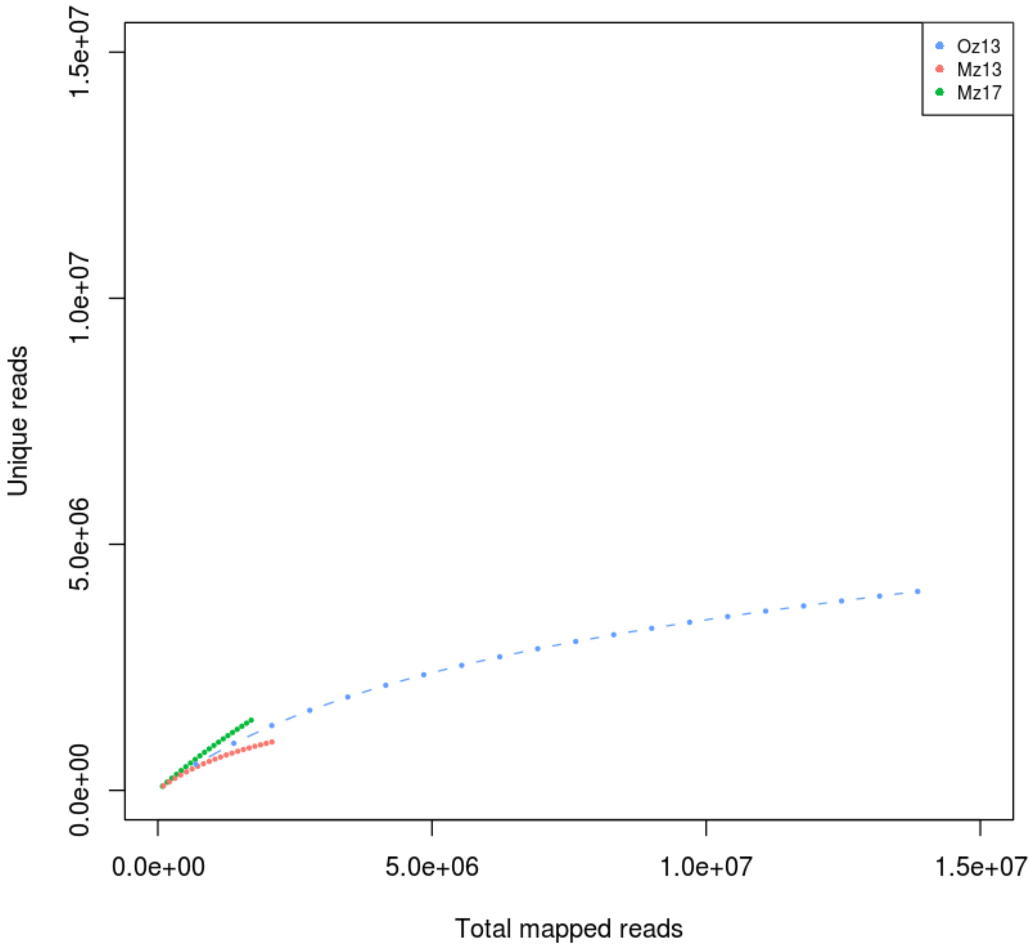

Figure 2: Unique reads per total mapped reads subsampling. Each point within the dotted lines indicates a 5% increase in the total number of the reads (X axis) for each sample. On the Y axis we show the proportion of unique reads mapped for each 5% increment.

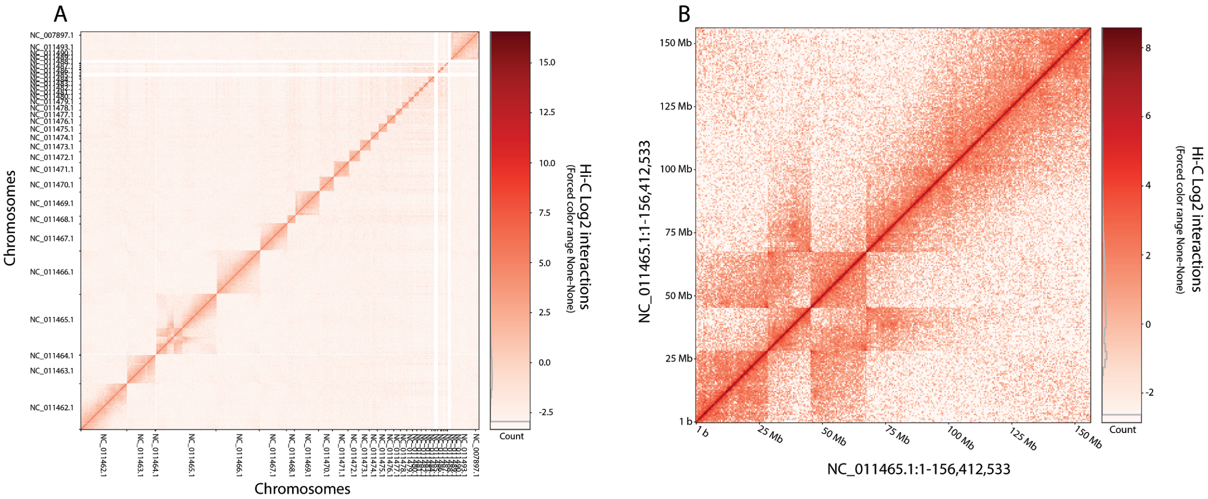

Figure 3: A) Hi-C contact matrix representation of the genome of sample Oz13. Note the presence of the rearrangement/translocations in chromosome NC_011465.1, as mentioned in the text.B) Close-up detail of the chromosome NC_011465, Vanilla normalized at a 500 kb resolution. Note the intrachromosomal rearrangements with relation to the reference genome.

Sample	Index	Read	Initial reads	% Digested sites (out of 100K reads)	% Reads with ligation site (out of 100K reads)	Uniquely mapped pairs (% initial)	Total # interactions; both reads mapped; (% initial)
Oz13	10	Read 1	29,436,352	83.6	31.6	18,670,087 (63.2%)	
		Read 2	29,436,352	80.4	29.8	18,127,333 (61.6%)	18,269,316 (62.1%)
Mz13	18	Read 1	3,069,136	68.5	14.8	2,396,713 (78.1%)	
		Read 2	3,069,136	66.2	14.4	2,297,654 (74.9%)	2,201,431 (71.7%)
Mz17	19	Read 1	2,274,286	44.8	7.8	1,711,896 (75.3%)	
		Read 2	2,274,286	42.5	7.3	1,673,397 (73.6%)	1,554,764 (68.4%)

Table 1. TADbit mapping and quality statistics of the Hi-C-BGI experimental results.

Filter type	Illumina ~expected % (for *MboI*)	Oz13	%	Mz13	%	Mz17	%
**Self-circle**	**<1%**	11,385	0.06	540	0.02	440	0.03
**Dangling-end**	**2.3 - 58.4%**	6,072,260	33.2	934,066	42.4	781,281	50.3
**Error**	**<1 - 3.4%**	8,941	0.05	586	0.02	587	0.03
**Extra dangling-end**	**9.5 - 70.1%**	5,275,752	28.9	744,126	33.8	517,306	33.3
**Too close from RES**	**23 - 93.7%**	5,348,693	29.3	642,844	29.2	221,954	14.3
**Too short**	**3.6 - 20%**	924,068	5	94,940	4.3	44,972	2.9
**Too large**	**<1 - 0.14%**	21	0.0001	1	0.000045	0	0
**Over-represented**	**3.16 - 13.2%**	3,362,668	18.4	446,571	20.3	64,224	4.1
**Duplicated**	**1.94 - 64.2%**	13,637,482	74.6	1,186,193	53.9	259,406	16.7
**Random breaks**	**<1 - 3.5%**	979,909	5.4	129,690	6.3	173,515	11.4
**Valid-pairs**	**7.5-98%**	4,043,904	22.1	848,181	38.5	1,117,826	71.7

Table 2: For each of the three experiments, reads lost after each of the filters applied, approximate comparison to numbers expected for an standard Illumina experiment, and final number of valid pairs considered. Note that the number of reads does not necessarily add up to the total number of interactions because a same read can be categorized within more than one of the filter categories. Valid pairs represents the number of reads used for generating the Hi-C maps seen in Figure 1.

Click here to access/download
Supplementary Material
SandovalVelasco_etal_GigascienceTN_SI.pdf
