# Supplementary material for: Hi-C chromosome conformation capture sequencing of avian genomes using the BGISEQ-500 platform
Source: Gigascience. 2020 Aug 26;9(8):giaa087. doi: 10.1093/gigascience/giaa087 (PMC7448675; doi:10.1093/gigascience/giaa087)
Supplement: giaa087_Supplemental_File [file giaa087_supplemental_file.docx]

**SUPPLEMENTARY INFORMATION**

**Hi-C chromosome conformation capture sequencing of avian genomes using the BGISEQ-500 platform**

Marcela Sandoval-Velasco^1*^, Juan Antonio Rodríguez^2*^, Cynthia Perez Estrada^3^, Guojie Zhang^4^, Erez Lieberman Aiden^3,5,6,7^, Marc A. Marti Renom^2,8,9,10^, M. Thomas P. Gilbert^1,11^, & Oliver Smith^1,12^

^1^ Section for Evolutionary Genomics, University of Copenhagen, 1353 Copenhagen, Denmark

^2^ CNAG-CRG, Centre for Genomic Regulation, Barcelona Institute of Science and Technology, 08028 Barcelona, Spain

^3^ Center for Genome Architecture, Department of Molecular and Human Genetics, Baylor College of Medicine, Houston TX, USA

^4^ China National GeneBank, BGI-Shenzhen, Shenzhen 518083, China

^5^ Center for Theoretical Biological Physics, Rice University, Houston TX, USA

^6^ Broad Institute of the Massachusetts Institute of Technology and Harvard University, Cambridge MA, USA

^7^ Department of Computer Science and Computational Applied Mathematics, Rice University, Houston TX, USA

^8^ Centre for Genomic Regulation, The Barcelona Institute for Science and Technology, Carrer del Doctor Aiguader 88, Barcelona, 08003, Spain

^9^ Pompeu Fabra University, Doctor Aiguader 88, Barcelona, 08003, Spain

^10^ ICREA, Pg. Lluís Companys 23, 08010 Barcelona, Spain

^11^ Norwegian University of Science and Technology, University Museum, 7491 Trondheim, Norway

^12^ Micropathology Ltd, University of Warwick Science Park, Coventry CV4 7EZ, UK

*M.S.-V and J.A.R contributed to this work equally

Corresponding authors:

marcela.velasco@sund.ku.dk

tgilbert@sund.ku.dk

oliver.smith@palaeome.org

**Supplementary Materials and Methods**

**Samples**

Three muscle tissue samples (Oz13, Mz13 and Mz17) were collected from two different zebra finch (*Taeniopygia guttata*) specimens (z13 and z17). Samples were coarsely crushed using a scalpel and the in-situ BGI-Hi-C protocol described below was followed.

**BGI-Hi-C protocol**

*Hi-C protocol adapted for BGI sequencing*

***Required oligos***

>AD1_short

AAGTCGGAGGCC

>AD1_long

TTGTCTTCCTAAGACCGCTTGGCCTCCGACTT

>AD2_short

AAGTCGGATCGT

>AD2_long

TTGTCTTCCTAAGGAACGACATGGCTACGATCCGACTT

>BGI_universal_primer (forward)

GAACGACATGGCTACGAT

>BGI_index1_reverse_primer (index sequence in **bold**)

TGTGAGCCAAGGAGTTG**ATTTATGACA**TTGTCTTCCTAAGACCGC

We recommend users contact BGI directly to discuss appropriate indexing combinations.

***Adapter preparation***

Begin by making a 500µM stock concentration of each adapter, to result in a 100µM universal adapter mix, and dilute as necessary. For this protocol, we used 3µl of a 4.5 µM dilution for the *Blunt-end Ligation step* (see step 62).

A. Prepare hybridisation buffer:

500mM NaCl

10mM Tris-HCl, pH 8.0

1mM EDTA, pH 8.0

B. Add the following to a 0.2ml low-bind PCR tube:

40µl of 500µM AD1_long

40µl of 500µM AD1_short

10µl hybridisation buffer

10µl molecular-grade water

C. Add the following to a separate 0.2ml low-bind PCR tube:

40µl of 500µM AD2_long

40µl of 500µM AD2_short

10µl hybridisation buffer

10µl molecular-grade water

D. Place both tubes into a thermal cycler. Heat to 95°C for 10 seconds, and cool to 12°C with a slow ramp of 0.1°C / second.

E. Combine both reactions into a 1.5ml low-bind eppendorf tube, for a ready-to-use 100µM adapter mix.

***Crosslinking***

1. Coarsely crush tissue material with scalpel. Particles of 0.1 – 1mm diameter should suffice. Transfer sample to 5ml low-bind eppendorf tubes.

2. Add in 500µL RPMI buffer to cover the sample.

3. Under a fume hood, add freshly made formaldehyde (crosslinking solution) to a final concentration of 1% v/v and mix gently.

4. Incubate at room temperature for 15 min with slow rotation/movement.

5. Add 2.5M glycine solution to a final concentration of 0.2M. Mix to quench reaction, dilute and wash away all remaining formaldehyde.

6. Vortex thoroughly. Incubate 5 min at RT with slow rotation/movement.

7. Centrifuge at 500 x *g* for 5 min. Discard supernatant into appropriate chemical waste.

8. Add 1000ml 1x PBS and mix to wash

9. Repeat step 6 and 7.

**SAFE STOP** Continue to lysis and digestion or store at -80ºC.

***Lysis and digestion***

*Note: lysis buffer should be made fresh. Reaction must be kept in ice/cold*

10. Prepare fresh lysis buffer by combining 10mM Tris.HCl pH 8, 10mM NaCl, 0.2% lgepal CA630 and 1X Protease inhibitors solution.

11. Grind the tissue with a pestle inside their tubes and then add 350µL of ice-cold lysis buffer to each sample. Buffer should cover all the sample

12. Resuspend the sample and let incubate on ice for 15 min

13. Centrifuge @ 2500g for 5 min

14. Discard supernatant without disrupting the pellet

15. Wash the pellet with 500µl of ice-cold lysis buffer

16. Centrifuge again @ 2500g for 5 min

17. Discard supernatant and add 100µL of 0.5% SDS

18. Incubate @ 62ºC for 10 min

*Note: Given the large amount of tissue being processed in the experiments reported in this article, for the next 10 steps (steps 20-30) we doubled the volume of reagents used in the original protocol by Rao et al., 2014.*

19. After incubation add 290µL of water and 50µL of 10% Triton X-100 to quench the SDS

20. Mix well and incubate for 15 min @ 37ºC

21. Add 50µL of 10X NEB Buffer 2 and 200U (8µL) of MboI restriction enzyme

22. Let digest overnight @ 37ºC with slow rotation/movement

***Biotin tagging***

23. Incubate at 62ºC for 10 min to inactivate MboI

24. Add the following to the reaction recovered from step 23:

· 75µl 0.4mM biotin-14-dCTP

· 3µl 10mM dATP

· 3µl 10mM dTTP

· 3µl 10mM dGTP

· 16µl DNA polymerase I, Large (Klenow) fragment

25. Mix well by flicking/pipetting and incubate for 1.5 hours at 37ºC with slow rotation/movement.

***Proximity ligation***

26. Prepare ligation Master Mix for the number of samples by adding 1800µL the following to the reaction:

· 1326µl water

· 240µl 10X NEB T4 ligase buffer

· 200µl 10% Triton X-100

· 24µl 10mg/ml BSA

· 1µl 400U/ml T4 ligase

27. Mix by inversion and incubate at room temperature for 4 hours with slow rotation/movement.

28. To degrade proteins add 150µl 20mg/ml proteinase K and 240µl 10% SDS.

29. Incubate overnight at 55 ºC with slow rotation/movement.

***DNA shearing***

30. Remove samples from incubation and let cool at RT

31. Spin samples for 5 min @ 2000xg

32. Transfer (**keep!**) supernatant to a new tube - DNA and ligated chromatin should be now in solution

33. Add 3X of 100% EtOH and 3µL of glycogen to each sample

34. Mix by inversion and leave for incubation at -80ºC for 15 min

35. Centrifuge at max speed for 20 min. Keep the tubes on ice after spinning and carefully remove supernatant by pouring out without disturbing the pellet.

36. Resuspend and wash the pellet with 1ml of 85% EtOH

37. Transfer sample suspension to new low-bind 1.5mL eppendorf tubes and centrifuge for 5 min at max speed

38. Remove all supernatant without disturbing the pellet. If necessary give a short spin to collect and remove all the EtOH without disturbing the pellet

39. By pipetting dissolve the pellet in 130µL of 1X Tris buffer (10mM Tris-HCl) and incubate for 10 min at 37ºC to fully dissolve the DNA

40. Transfer sample to a Covaris 130µL microtube and shear DNA to 300-500 bp

41. Transfer sheared DNA to new low-bind 1.5mL eppendorf tubes and collect all DNA from the Covaris vial by washing with 70μl of water and add to the sample, bringing the total reaction volume to 200μl.

**SAFE STOP** Continue to size selection or biotin pull-down, or store at -20ºC.

*Note: The original protocol by Rao et al., (2014) includes a size selection step. In this protocol we skipped that step to avoid the risk of DNA loss.*

***Size selection*** *(optional)*

42. Warm a bottle of AMPure XP beads (Beckman Coulter, A63881) to room temperature before performing the double size selection.

43. Add 0.55X volumes of beads to the reaction. Mix well by pipetting and incubate at room temperature for 5 minutes.

44. Separate on a magnet. Transfer the clear solution to a fresh tube, avoiding any beads. The supernatant will contain fragments shorter than 500bp.

45. Add exactly 30μl of fresh AMPure XP beads to the solution. Mix by pipetting and incubate at room temperature for 5 minutes.

46. Separate on a magnet and keep the beads. Fragments in the range of 300-500bp will be retained on the beads. Discard the supernatant containing degraded RNA and short DNA fragments.

47. Keeping the beads on the magnet, wash twice with 700μl of 70% ethanol without mixing.

48. Leave the beads on the magnet for 5 minutes to allow remaining ethanol to evaporate.

49. To elute DNA, add 300μl of 1X Tris buffer, gently mix by pipetting, incubate at room temperature for 5  minutes, separate on a magnet, and transfer the solution to a new low-bind 1.5mL eppendorf tube.

***Biotin Pull-Down***

Perform all the following steps in low-bind tubes.

50. Prepare for biotin pull-down by washing 100μl of 10mg/ml Dynabeads MyOne Streptavidin T1 beads (Life technologies, 65602) with 400μl of 1X Tween Washing Buffer (1X TWB: 5mM Tris-HCl (pH 7.5); 0.5mM EDTA; 1M NaCl; 0.05% Tween 20). Separate on a magnet and discard the solution.

51. Resuspend the beads in 300μl of 2X Binding Buffer (2X BB: 10mM Tris-HCl (pH 7.5); 1mM EDTA; 2M NaCl) and add to the reaction. Incubate at room temperature for 15 minutes with rotation to bind biotinylated DNA to the streptavidin beads.

52. Separate on a magnet and discard the supernatant.

53. Wash the beads by adding 600μl of 1X TWB and incubate the tubes on a Thermomixer at 55°C for 2 min with mixing (600rpm). Reclaim the beads using a magnet. Discard supernatant.

55. Repeat wash.

55. Resuspend beads in 100ul 1X NEB T4 DNA ligase buffer (NEB, B0202) Separate on a magnet to reclaim beads and discard the supernatant.

***Preparation for BGI Sequencing***

***End Repair -*** repair ends of sheared DNA and remove biotin from unligated ends

56. Resuspend beads in 100μl of End Repair master mix:

· 88μl of 1X NEB T4 DNA ligase buffer with 10mM ATP (NEB, B0202)

· 2μl of 25mM dNTP mix

· 5μl of 10U/μl NEB T4 PNK (NEB, M0201)

· 4μl of 3U/μl NEB T4 DNA polymerase I (NEB, M0203)

· 1μl of 5U/μl NEB DNA polymerase I, Large (Klenow) Fragment (NEB, M0210)

57. Incubate at room temperature for 30 minutes. Separate on a magnet to reclaim beads and discard the supernatant.

58. Wash the beads by adding 600μl of 1X TWB. Incubate the tubes on a Thermomixer at 55°C for 2 min with mixing (600rpm). Separate on a magnet to reclaim beads and discard the supernatant.

59. Repeat wash.

***Blunt-end Ligation***

60. Resuspend beads in 100μl 1X Tris Buffer and transfer to a new tube.

61. Separate on a magnet to reclaim beads, discard the supernatant and add 50µL of 1X NEB Quick Ligation Buffer to resuspend the beads. Then add 2µL of DNA Quick Ligase and 3µL of BGI adapter mix (final concentration 0.25µM) to each sample.

62. Incubate 15 min at RT.

63. Separate on a magnet to reclaim beads and discard the supernatant

64. Wash the beads by adding 600μl of 1X TWB. Incubate the tubes on a Thermomixer at 55°C for 2 min with mixing (600rpm). Separate on a magnet to reclaim beads and discard the supernatant.

65. Repeat wash.

66. Resuspend beads in 100μl 1X Tris Buffer. Separate on a magnet to reclaim beads and discard the supernatant.

***Adapter fill-in***

BGI adapters contain distal overhangs, the missing bases forming the priming site for indexing PCR. Therefore an additional adapter fill-in step is required. For simplicity, repeat “***End repair***” and proceed to ***qPCR*** or ***PCR index amplification*.**

67. Resuspend beads in 100μl of End Repair master mix:

· 88μl of 1X NEB T4 DNA ligase buffer with 10mM ATP (NEB, B0202)

· 2μl of 25mM dNTP mix

· 5μl of 10U/μl NEB T4 PNK (NEB, M0201)

· 4μl of 3U/μl NEB T4 DNA polymerase I (NEB, M0203)

· 1μl of 5U/μl NEB DNA polymerase I, Large (Klenow) Fragment (NEB, M0210)

68. Incubate at room temperature for 30 minutes. Separate on a magnet to reclaim beads and discard the supernatant.

69. Wash the beads by adding 600μl of 1X TWB. Incubate the tubes on a Thermomixer at 55°C for 2 min with mixing (600rpm). Separate on a magnet to reclaim beads and discard the supernatant.

70. Repeat wash and resuspend beads in 50µL of 1X Tris Buffer.

***qPCR and PCR index amplification***

71. To estimate optimal number of amplification cycles for each sample, run a 20µL qPCR reaction using 2µL of a 1:10 dilution of BGI-Hi-C library as template.

72. Amplify on-bead using BGI forward and reverse_index primers and KAPA HiFi Ready Mix (KR0368) or Phusion High Fidelity PCR Master Mix (F531L).

*Note: when working with non-optimal material, extra PCR cycles may be necessary due to*

*the number of wash cycles that makes this protocol somehow inefficient.*

73. After amplification, clean up PCR products with your preferred method (AmpureXP / SPRI beads / MinElute columns) depending on whether size selection is necessary.

74. Quantify in Qubit and TapeStation or BioAnalyzer.

75. Continue with PE sequencing on a BGISEQ platform.

**Supplementary Figures**

**
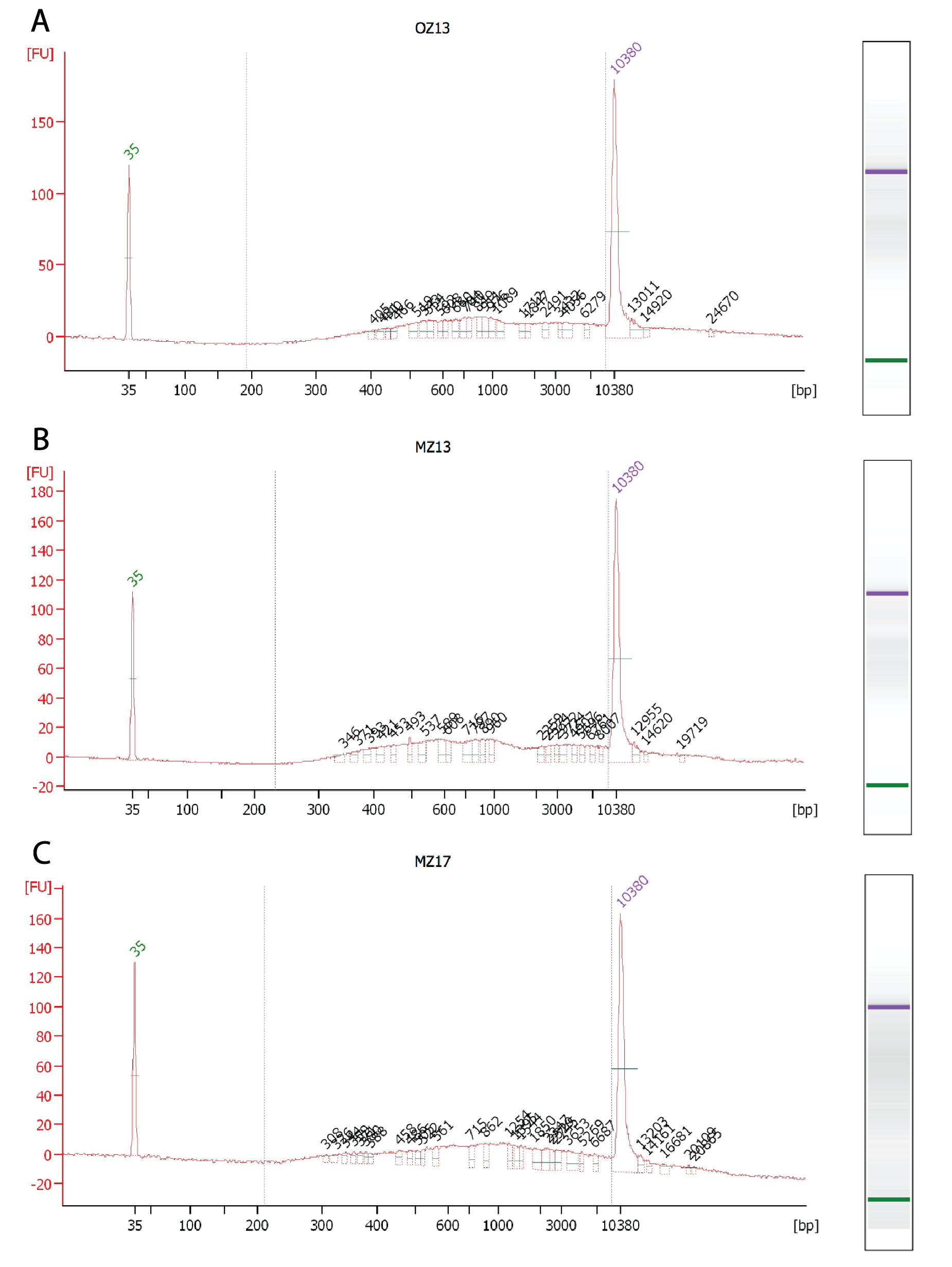
**

**Figure S.1:** BioAnalyzer trace images for the three samples showing the fragment length distribution of DNA recovered after amplification of the BGI-Hi-C library.

**Figure S.2:** Insert size plots for the three samples - A) Oz13, B) Mz13 and C) Mz17 - showing the fragment length distribution of the sequenced DNA fragments.

**
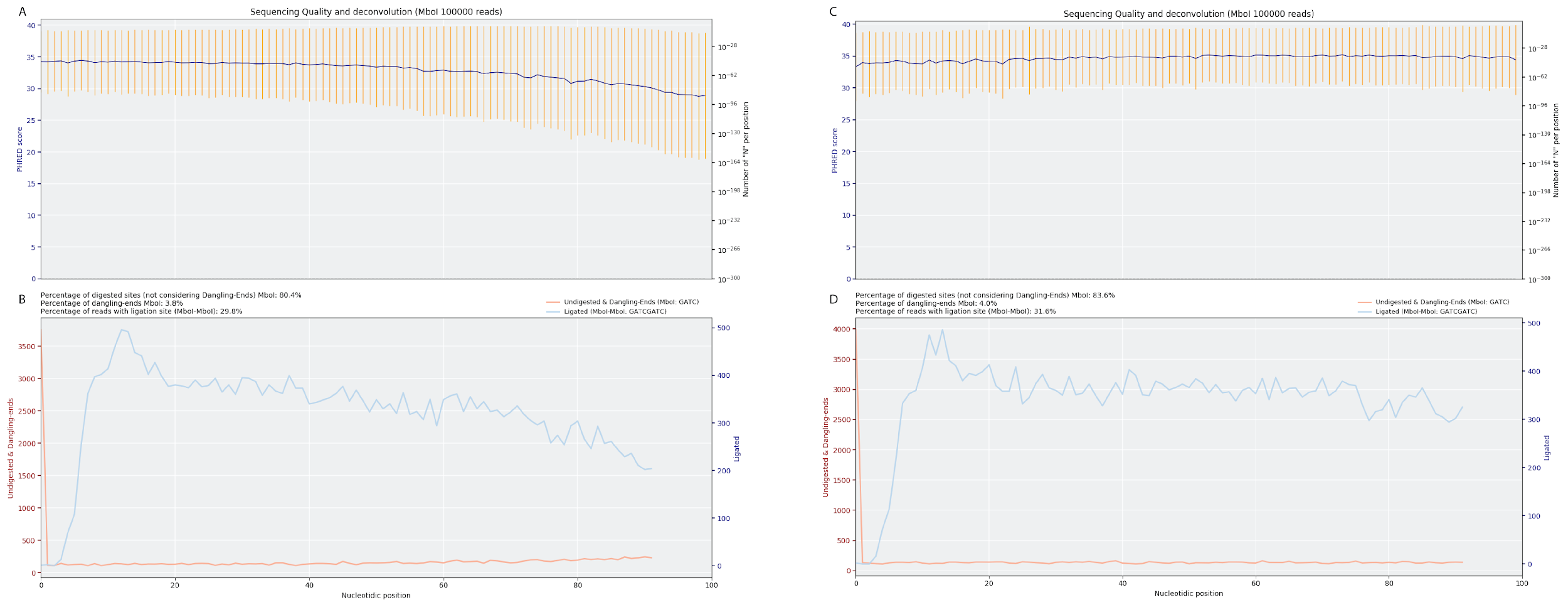
**

**Figure S.3:** Quality assessment of the Hi-C experiment for sample Oz13 before mapping to the reference genome. Plots (A and C) represent the per nucleotide quality profile of the sequencing reads based on the PHRED score, including the proportion of unidentified nucleotides (Ns) found at each position. Plots (B and D) show the proportion of reads with ligation sites as well as the number of reads starting by a cut-site, a proxy to first assess the efficiencies of the digestion and the ligation in the HiC experiment. Plots A and B correspond to read 1 and plots C and D correspond to read 2.


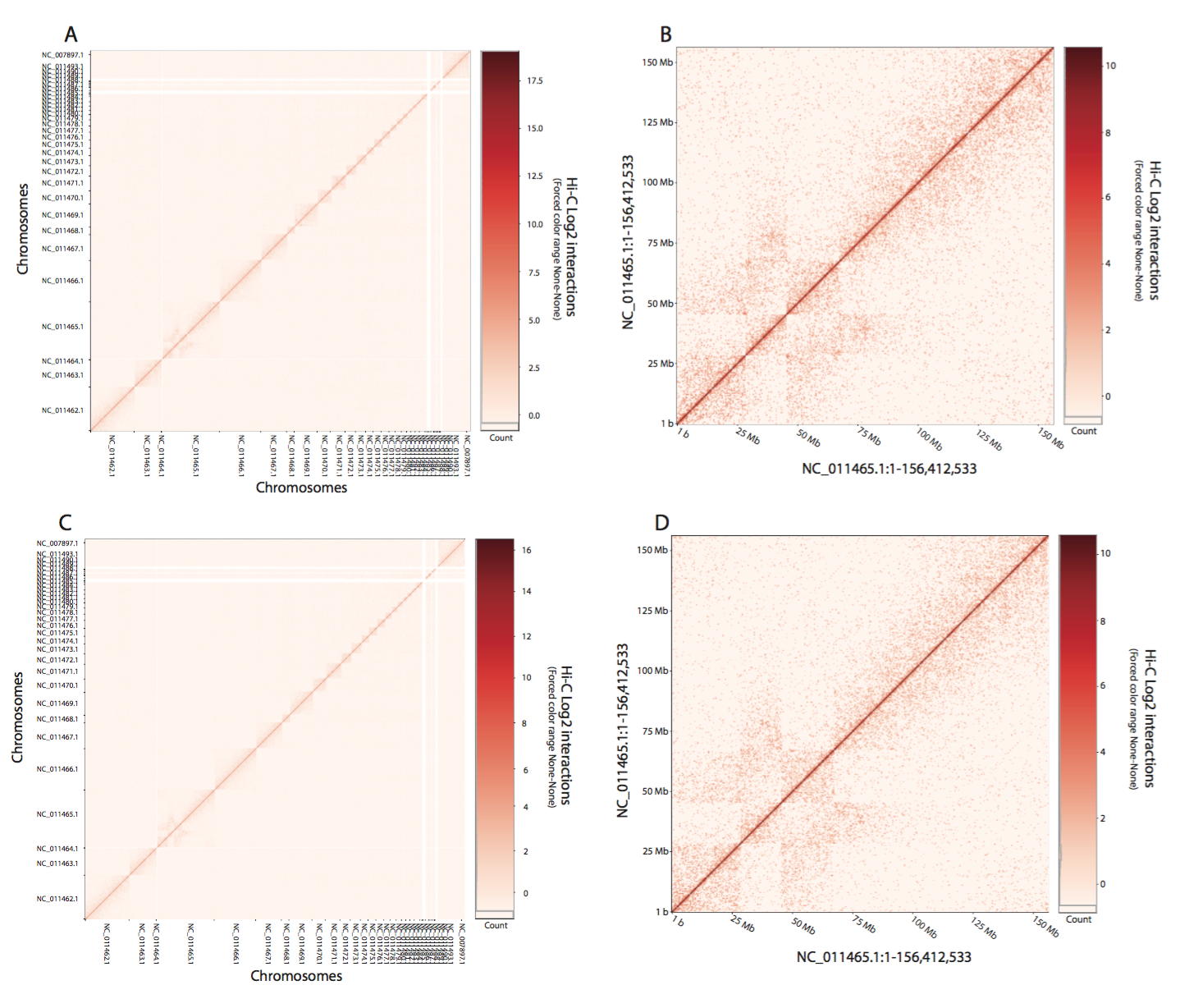


**Figure S.4:** Hi-C contact matrix representation of the genome of samples Mz13 (A and B) and Mz17 (C and D) at a resolution of 500 kb. Panels A and C are a representation of the whole genome, which panels B and D are a close-up representation of the chromosome NC_011465.
