# Supplementary material for: Hi-C chromosome conformation capture sequencing of avian genomes using the BGISEQ-500 platform
Source: Gigascience. 2020 Aug 26;9(8):giaa087. doi: 10.1093/gigascience/giaa087 (PMC7448675; doi:10.1093/gigascience/giaa087)
Supplement: giaa087_Response_to_Reviewer_Comments_Original_Submission [file giaa087_response_to_reviewer_comments_original_submission.pdf]

Author's Response To Reviewer Comments

Close

Response to Reviewers

Reviewer #1: Sandoval-Velasco M, Rodriguez JA, et al presented an interesting manuscript on comparison between the established Illumina sequencing platform and novel BGISEQ in application for Hi-C analysis. The state of the art is well reviewed and the case for the importance of this is well made. As the authors highlighted, there is an urgent need to explore other sequencing chemistries, alternative to Illumina platforms, to introduce competition into this market and reduce costs. The topic is novel and to my knowledge has not been investigated elsewhere. Although, BGISEQ technology has been validated (RNA-seq, exon-seq) or used (ATAC-seq, ChiP-seq…) for other "omics", 3C-based methods remains unexplored. For these reasons, this study adds an interesting perspective for future studies of the spatial-temporal genome architecture. The current version of manuscript is well written, concise and its conclusions are both reasonable and well founded. However, a haave some comments with the aim to improve this manuscript:
REPLY - We appreciate the reviewers' opinion and the time spent reviewing our manuscript. We hope our study will indeed allow more researchers to explore questions related to the spatial-temporal genome architecture and expand their sequencing choices.

Major comments
- I am a little bit concerned about the quality of the Hi-C libraries and its effect on the further analysis. Hi-C libraries are not very complex (they reach saturation sequencing around 18 millions reads) and the size distributions (Bioanalyzed profiles) do not follow the criteria of Illumina or BGI.
REPLY - We are a little confused about the meaning of the reviewers' comment on this point, and would appreciate if she/he could elaborate if our reply does not suffice. The molecular complexity estimated as the number of unique reads per total mapped reads (Figure 2) varies per sample as is expected in Hi-C experiments. The Bioanalyzer profiles, show longer tails than expected, this may be because of skipping the last size selection step, which we consider optional given it results in the loss of a lot of DNA. To clarify this point we have included a new figure in the supplementary (Figure S.2) showing the fragment length distribution of the sequenced DNA fragments. Based on our experience these profiles are acceptable for Hi-C experiments. We have added the reference to this new figure on line 136 of the main text.

- In addition, they only generate one biological replicate, which does not allow addressing reproducibility. For this reason, it would be appreciate if the authors could generate a second biological replicate with higher quality. Correlation and clustering analysis between all 6 biological replicates, as well as with the 320 in situ Hi-C experiments available at the repository of the 4DGenome unit at the CRG, could provide fundamental insights to further extend their conclusions. In addition, despite of the very valuable use of a broad collection of 320 Hi-C dataset, the genome architecture of zebra finch has not been study on the Illumina platform. For this reason, I recommend to adapt and sequence by the Illumina chemistry at least one of these 6 Hi-C libraries. All these recommendations are suggestions, and the editor should take the current worldwide scenario because of the Coronavirus outbreak in consideration.
REPLY - We appreciate the reviewers' comment and we acknowledge that performing several replicate experiments for each sample would be the optimal. We explored the possibility of doing so, but given the challenging times we are all facing due to COVID19 lab lockdowns we are unable to do this at the moment. However, ultimately, we see our study as a proof of principle to showcase the availability of BGISEQ platforms to sequence Hi-C experiments, and we hope that by showing that is possible, other researchers will start considering it as it also represents a more cost competitive platform for this purpose.
We are not 100% sure we understand the reviewer's comment with regards to the correlation and clustering analysis suggested, and would appreciate if she/he could elaborate so we could address it accordingly.

Minor comments.

- The BGI technology is based on iterative ligations to circularise the DNA molecules, followed by amplification. Please, clarify it at the background section. It is a little bit confusing the manner in which they explain it, and could be interpreted as three steps: ligation, circularisation and amplification.
REPLY - We have modified the text based on the reviewers' suggestion. The description of the BGISEQ technology starting on line 102, now reads:
"... the BGISEQ technology combines DNA nanoball nanoarrays [14] with polymerase- based stepwise sequencing. During this process, also called nanoball sequencing, the DNA undergoes an iterative ligation to circularize the DNA molecules, which are then replicated for the generation of DNA nanoballs. This iterative process generates billions of DNA nanoballs from each DNA molecule that are then loaded into a flow cell and sequenced [15]."

- Please, indicate the estimation of cell numbers processed in each Hi-C biological replicate and the amount of starting tissue.
REPLY - Following the recommendation, we have indicated the amount of tissue used for each Hi-C biological replicate in the Methods description, line number 187 of the main manuscript. Unfortunately we are unable to provide an estimation of cell numbers processed in each, given that we didn't perform microscopy experiments on the tissue samples.

- Please, indicate with an arrow the breakpoints to help non-expert readers at the Figure 3.
REPLY - We have added coloured arrows to Figure 3 as suggested by the reviewer.

Reviewer #2: The paper "Hi-C chromosome conformation capture sequencing of avian genomes using the
BGISEQ-500 platform" by Marcela Sandoval-Velasco et al describes a new apply BGISEQ-500 platform to Hi-C technique. Recently, Hi-C analysis broadly used for genome scale study, but Illumina-based sequencing was the only way to make a connection. In this situation, this technical paper has a good advantage in that BGISEQ platform provides another choice. Unfortunately, Mz13 and Mz17 samples were too small amount of data, so these did not fully described this methods are relevantly appreciated. Despite these, Proven technique will provide a good protocol for the research of HiC analysis.
REPLY - Thank you for your comment. We appreciate you appreciate the fact a method has been developed that will help others in their research.

Reviewer #3: This is a very nice technical note that I think will be incredibly useful for the field. I hope that it will help in democratizing sequencing costs across the scientific space where Illumina is being challenged by BGI. I find more or less no issues, other than a few minor comments below. I also will apologize for how long it took me to get this review back to you--it was a pleasure to read the note. I hope also that the authors are staying safe and well during these times.
REPLY - We thank the reviewer for her/his comments and appreciation of our study. We also hope this helps the development of the field and allows a growing number of researchers to explore and apply these new techniques on their research.

Intro:
Albeit this is a technical note, I think most of the field is not very familiar with BGI. You do a nice job describing how the tech at the chemistry level is different, but it might be good to include some basics in how it compares on read length etc --know the papers are cited but just a sentence or two might be good. You say later you use 100PE, but would be nice in the intro when you are comparing the platforms.
REPLY - Following the reviewers' suggestion, we have expanded the description of the BGISEQ platform specifics from line 102 in the main manuscript. The new sentences now read:
"... the BGISEQ technology combines DNA nanoball nanoarrays [14] with polymerase- based stepwise sequencing. During this process, also called nanoball sequencing, the DNA undergoes an iterative ligation to circularize the DNA molecules, which are then replicated for the generation of DNA nanoballs. This iterative process generates billions of DNA nanoballs from each DNA molecule that are then loaded into a flow cell and sequenced[15]. The BGISEQ has several features that have proven attractive to researchers within different fields. Namely, it allows several sequencing read-lengths (50-100-150bp) either for single read [SR] or paired end [PE] sequencing; it has a very high throughput where at least 2 billion PE reads per flow cell are generated in only a few days; and it allows an easy adaptation of library construction protocols."

Methods:
Could you maybe add the motivation in for skipping size selection in your protocol? Most of your other steps have nice motivating sentences behind them for the protocol modifications you made.
--Now I see this is in the supplement, I would move this into the main. It's only one sentence but as someone who has done a lot of HiC prep, I was confused that this step was left out.
REPLY - We have addressed the reviewers' comment and added this information in the Methods description part of the main text, line 191. The new sentence now reads:
"... To avoid the risk of DNA loss we skipped the size selection step described in the original protocol, and continued with preparing our samples for BGI sequencing."

Tables and figures:
Perhaps switch the color of your dots to be color blind friendly in figures one and two? Just the red and green.
REPLY - We thank the reviewer for noting the color scheme was not color blind friendly as we had missed this important point. Following the suggestion, we have accordingly modified both figures 1 and 2.

Figure 1: A few q's --is the blue dot covered up for the "too close from res?". Based on the 320 other experiments and where these samples place it seems unlikely that Oz13 didn't come up in this category?
REPLY - The sample point for Oz13 in the plot was overlapping with Mz13 (they have very close values: 0.2920 and 0.2927, respectively), so the points were plotted one over the other. This has been corrected now: points were "jittered" and now we make all of them visible.

Supplement:
General question--i know you commented on the source of the tissue, but it's my understanding that Hi-C can be quite sensitive to stored or older specimens. Were the 320 samples and these ones representative of a range of storage conditions/collection times? Perhaps that is driving some of the patterns you see? (Sure you have thought of this, but just curious)
REPLY - All of the 320 samples come from in-situ Hi-C experiments performed in-house at the CRG by four different persons at different time points. Samples were processed using 4-cutter enzymes (either MboI or DpnII, both cutting through the same 4-letter sequence) and sequenced within CRG facilities using Illumina platforms, either using Hi-Seq or Hi-Seq2000 machines (PE75).
As the reviewer states, the range of conditions for the 320 samples varies widely. Experiments were done in cell lines derived either from Homo sapiens (~68%) or Mus musculus (~32%). Cell types comprised: B-cells (at mature and precursor stages) and embryonic stem cells up to a ~50% of the samples, and the remaining ~50% were Hi-C experiments performed in commercial cancer cell lines, mostly from breast cancer, endometrial cancer and leukemia. Most of the samples (>75%) were untreated cells. Once the data were sequenced, all of the samples were processed using TADbit software, the same we use for the present study. In contrast, the three zebra finch samples we present on our study are based on fresh tissue samples collected and then frozen, instead of cell lines.
We do not believe that Hi-C final statistics could be strongly affected by just a single characteristic of the cell lines. If any, probably batch effects, precision errors, personalized protocols, noise, slight custom modifications from each experimenter or, most likely, any combination of these or other factors could affect the statistics we are seeing for the ensemble of 320 samples. Trying to identify these factors would be an interesting matter for a different paper, thus we think that it might be out of scope for the present paper.
With the present analysis we would like to convey the idea that the results we obtain just fit reasonably well within a range of possible outcomes of Hi-C. Actually, we do not observe any of our samples to be a huge outlier in any critical category, like errors or self-circles. Maybe for the random breaks category we see in our samples rise up to a 6-10%, but this is directly related to the sample type, the storage conditions, age, or the amount of degradation of DNA, and not directly related to the protocol itself (computationally or experimentally).
All these results emphasize the idea of BGI as an alternative and reliable approach for Hi-C experiments.
We would like to note that we removed samples that contained at least 1 NA value for any of the filter categories in Figure 1. Applying this, we removed 4 samples, so the final number now is 316, instead of 320. This does not affect any conclusions or results. This is corrected now in the main text and Supplementary Information.

I am impressed by the detail and ease that it would be to follow the protocol you have outlined here.
REPLY - We appreciate this positive comment and hope that many researchers will make use of the protocol and method we describe in our study.

Close
