# Supplementary material for: Hi-C chromosome conformation capture sequencing of avian genomes using the BGISEQ-500 platform
Source: Gigascience. 2020 Aug 26;9(8):giaa087. doi: 10.1093/gigascience/giaa087 (PMC7448675; doi:10.1093/gigascience/giaa087)
Supplement: giaa087_Reviewer_1_Report_Original_Submission — Biola M. Javierre -- 4/6/2020 Reviewed [file giaa087_reviewer_1_report_original_submission.pdf]

Reviewer Report

Title: Hi-C chromosome conformation capture sequencing of avian genomes using the BGISEQ-500 platform

Version: Original Submission **Date:** 4/6/2020

Reviewer name: Biola M. Javierre

Reviewer Comments to Author:

Sandoval-Velasco M, Rodriguez JA, et al presented an interesting manuscript on comparison between the established Illumina sequencing platform and novel BGISEQ in application for Hi-C analysis. The state of the art is well reviewed and the case for the importance of this is well made. As the authors highlighted, there is an urgent need to explore other sequencing chemistries, alternative to Illumina platforms, to introduce competition into this market and reduce costs. The topic is novel and to my knowledge has not been investigated elsewhere. Although, BGISEQ technology has been validated (RNA-seq, exon-seq) or used (ATAC-seq, ChiP-seq…) for other "omics", 3C-based methods remains unexplored. For these reasons, this study adds an interesting perspective for future studies of the spatial-temporal genome architecture. The current version of manuscript is well written, concise and its conclusions are both reasonable and well founded. However, a have some comments with the aim to improve this manuscript:

Major comments

- I am a little bit concerned about the quality of the Hi-C libraries and its effect on the further analysis. Hi-C libraries are not very complex (they reach saturation sequencing around 18 millions reads) and the size distributions (Bioanalyzed profiles) do not follow the criteria of Illumina or BGI. In addition, they only generate one biological replicate, which does not allow addressing reproducibility. For this reason, it would be appreciate if the authors could generate a second biological replicate with higher quality. Correlation and clustering analysis between all 6 biological replicates, as well as with the 320 in situ Hi-C experiments available at the repository of the 4DGenome unit at the CRG, could provide fundamental insights to further extend their conclusions. In addition, despite of the very valuable use of a broad collection of 320 Hi-C dataset, the genome architecture of zebra finch has not been study on the Illumina platform. For this reason, I recommend to adapt and sequence by the Illumina chemistry at least one of these 6 Hi-C libraries. All these recommendations are suggestions, and the editor should take the current worldwide scenario because of the Coronavirus outbreak in consideration.

Minor comments.

- The BGI technology is based on iterative ligations to circularise the DNA molecules, followed by amplification. Please, clarify it at the background section. It is a little bit confusing the manner in which they explain it, and could be interpreted as three steps: ligation, circularisation and amplification.
- Please, indicate the estimation of cell numbers processed in each Hi-C biological replicate and the amount of starting tissue.
- Please, indicate with an arrow the breakpoints to help non-expert readers at the Figure 3.

Level of Interest

Please indicate how interesting you found the manuscript: Choose an item.

Quality of Written English

Please indicate the quality of language in the manuscript: Choose an item.

Declaration of Competing Interests

Please complete a declaration of competing interests, considering the following questions:

- Have you in the past five years received reimbursements, fees, funding, or salary from an organisation that may in any way gain or lose financially from the publication of this manuscript, either now or in the future?
- Do you hold any stocks or shares in an organisation that may in any way gain or lose financially from the publication of this manuscript, either now or in the future?
- Do you hold or are you currently applying for any patents relating to the content of the manuscript?
- Have you received reimbursements, fees, funding, or salary from an organization that holds or has applied for patents relating to the content of the manuscript?
- Do you have any other financial competing interests?
- Do you have any non-financial competing interests in relation to this paper?

If you can answer no to all of the above, write 'I declare that I have no competing interests' below. If your reply is yes to any, please give details below.

I declare that I have no competing interests

I agree to the open peer review policy of the journal. I understand that my name will be included on my report to the authors and, if the manuscript is accepted for publication, my named report including any attachments I upload will be posted on the website along with the authors' responses. I agree for my report to be made available under an Open Access Creative Commons CC-BY license (http://creativecommons.org/licenses/by/4.0/). I understand that any comments which I do not wish to be included in my named report can be included as confidential comments to the editors, which will not be published.

Choose an item.

To further support our reviewers, we have joined with Publons, where you can gain additional credit to further highlight your hard work (see: https://publons.com/journal/530/gigascience). On publication of this paper, your review will be automatically added to Publons, you can then choose whether or not to claim your Publons credit. I understand this statement.

Yes Choose an item.
