# Supplementary material for: Hi-C chromosome conformation capture sequencing of avian genomes using the BGISEQ-500 platform
Source: Gigascience. 2020 Aug 26;9(8):giaa087. doi: 10.1093/gigascience/giaa087 (PMC7448675; doi:10.1093/gigascience/giaa087)
Supplement: giaa087_Reviewer_3_Report_Original_Submission — Ellie Armstrong -- 4/30/2020 Reviewed [file giaa087_reviewer_3_report_original_submission.pdf]

Reviewer Report

Title: Hi-C chromosome conformation capture sequencing of avian genomes using the BGISEQ-500 platform

Version: Original Submission **Date:** 4/30/2020

Reviewer name: Ellie Armstrong

Reviewer Comments to Author:

This is a very nice technical note that I think will be incredibly useful for the field. I hope that it will help in democratizing sequencing costs across the scientific space where Illumina is being challenged by BGI. I find more or less no issues, other than a few minor comments below. I also will apologize for how long it took me to get this review back to you--it was a pleasure to read the note. I hope also that the authors are staying safe and well during these times.
Intro:
Albeit this is a technical note, I think most of the field is not very familiar with BGI. You do a nice job describing how the tech at the chemistry level is different, but it might be good to include some basics in how it compares on read length etc --know the papers are cited but just a sentence or two might be good. You say later you use 100PE, but would be nice in the intro when you are comparing the platforms.
Methods:
Could you maybe add the motivation in for skipping size selection in your protocol? Most of your other steps have nice motivating sentences behind them for the protocol modifications you made.
--Now I see this is in the supplement, I would move this into the main. It's only one sentence but as someone who has done a lot of HiC prep, I was confused that this step was left out.
Tables and figures:
Perhaps switch the color of your dots to be color blind friendly in figures one and two? Just the red and green.
Figure 1: A few q's --is the blue dot covered up for the "too close from res?". Based on the 320 other experiments and where these samples place it seems unlikely that Oz13 didn't come up in this category?
Supplement:
General question--i know you commented on the source of the tissue, but it's my understanding that Hi-C can be quite sensitive to stored or older specimens. Were the 320 samples and these ones representative of a range of storage conditions/collection times? Perhaps that is driving some of the patterns you see? (Sure you have thought of this, but just curious)
I am impressed by the detail and ease that it would be to follow the protocol you have outlined here.

Level of Interest

Please indicate how interesting you found the manuscript: Choose an item.

Quality of Written English

Please indicate the quality of language in the manuscript: Choose an item.

Declaration of Competing Interests

Please complete a declaration of competing interests, considering the following questions:

- Have you in the past five years received reimbursements, fees, funding, or salary from an organisation that may in any way gain or lose financially from the publication of this manuscript, either now or in the future?
- Do you hold any stocks or shares in an organisation that may in any way gain or lose financially from the publication of this manuscript, either now or in the future?
- Do you hold or are you currently applying for any patents relating to the content of the manuscript?
- Have you received reimbursements, fees, funding, or salary from an organization that holds or has applied for patents relating to the content of the manuscript?
- Do you have any other financial competing interests?
- Do you have any non-financial competing interests in relation to this paper?

If you can answer no to all of the above, write 'I declare that I have no competing interests' below. If your reply is yes to any, please give details below.

I declare that I have no competing interests.

I agree to the open peer review policy of the journal. I understand that my name will be included on my report to the authors and, if the manuscript is accepted for publication, my named report including any attachments I upload will be posted on the website along with the authors' responses. I agree for my report to be made available under an Open Access Creative Commons CC-BY license (http://creativecommons.org/licenses/by/4.0/). I understand that any comments which I do not wish to be included in my named report can be included as confidential comments to the editors, which will not be published.

Choose an item.

To further support our reviewers, we have joined with Publons, where you can gain additional credit to further highlight your hard work (see: https://publons.com/journal/530/gigascience). On publication of this paper, your review will be automatically added to Publons, you can then choose whether or not to claim your Publons credit. I understand this statement.

Yes Choose an item.
